# Supplementary material for: Chromosomes of Asian cyprinid fishes: Novel insight into the chromosomal evolution of Labeoninae (Teleostei, Cyprinidae)
Source: PLoS One. 2024 Feb 7;19(2):e0292689. doi: 10.1371/journal.pone.0292689 (PMC10849230; doi:10.1371/journal.pone.0292689)
Supplement: Attachment — Submitted filename: PONE-D-23-14589 copy.pdf [file pone.0292689.s002.pdf]

PLOS ONE

Chromosomes of Asian cyprinid fishes: Novel insight into the chromosomal evolution of Labeoninae (Teleostei, Cyprinidae)

--Manuscript Draft--

**Manuscript Number:**	PONE-D-23-14589
**Article Type:**	Research Article
**Full Title:**	Chromosomes of Asian cyprinid fishes: Novel insight into the chromosomal evolution of Labeoninae (Teleostei, Cyprinidae)
**Short Title:**	Novel insight into the chromosomal evolution of Labeoninae (Teleostei, Cyprinidae)
**Corresponding Author:**	Weerayuth Supiwong, Ph.D.<br>Khon Kaen University<br>Nong Khai, THAILAND
**Keywords:**	Fish cytogenetics; karyotype evolution and stasis; FISH; repetitive DNAs
**Abstract:**	The Labeoninae subfamily is a highly diversified but demonstrably monophyletic lineage of cyprinid fishes comprising five tribes and six incertae sedis genera. This widely distributed assemblage contains some 48 genera and around 480 recognized species distributed in freshwaters of Africa and Asia. In this study, the karyotypes and other chromosomal properties of five Labeoninae species found in Thailand Labeo chrysophekadion (Labeonini) and Epalzeorhynchos bicolor, E. munense, Henicorhynchus siamensis, Thynnichthys thynnoides (´Osteochilini´) were examined using conventional and molecular cytogenetic protocols. Our results confirmed a diploid chromosome number (2n) invariably 2n = 50, but the ratio of uni- and bi-armed chromosomes was highly variable among their karyotypes, indicating extensive structural chromosomal rearrangements. Karyotype of L. chrysophekadion contained 10m+6sm+20st+14a, 32m+10sm+8st for H. siamensis, 20m+12sm+10st+8a in E. bicolor, 20m+8sm+8st+14a in E. munense, and 18m+24sm+8st in T. thynnoides. Except for H. siamensis, which had four sites of 5S rDNA sites, other species under study had only one chromosome pair with those sites. In contrast, only one pair containing 18S rDNA sites were found in the karyotypes of three species, whereas two sites were found in that of E. bicolor. These cytogenetic patterns indicated that the cytogenomic divergence patterns of these labeonine species largely corresponded to the inferred phylogenetic tree. In spite the 2n stability, diverse patterns of rDNA and microsatellite distribution as well as their various karyotype structures demonstrated significant evolutionary differentiation of Labeoninae genomes as exemplified in examined species.
**Order of Authors:**	Sudarat Khensuwan
	Francisco de Menezes Cavalcante Sassi, Master
	Renata Luiza Rosa de Moraes
	Petr Rab
	Thomas Liehr
	Weerayuth Supiwong, Ph.D.
	Kriengkrai Seetapan
	Alongklod Tanomtong
	Nathpapat Tantisuwichwong
	Satit Arunsang
	Phichaya Buasriyot
	Sampun Tongnunui
	Marcelo de Bello Cioffi, PhD
**Additional Information:**	

Powered by Editorial Manager® and ProduXion Manager® from Aries Systems Corporation

Question	Response
**Financial Disclosure**<br><br>Enter a financial disclosure statement that describes the sources of funding for the work included in this submission. Review the submission guidelines for detailed requirements. View published research articles from *PLOS ONE* for specific examples.<br><br>This statement is required for submission and **will appear in the published article** if the submission is accepted. Please make sure it is accurate.<br><br>**Unfunded studies**<br>Enter: *The author(s) received no specific funding for this work.*<br><br>**Funded studies**<br>Enter a statement with the following details:<br>• Initials of the authors who received each award<br>• Grant numbers awarded to each author<br>• The full name of each funder<br>• URL of each funder website<br>• Did the sponsors or funders play any role in the study design, data collection and analysis, decision to publish, or preparation of the manuscript?<br>• **NO** - Include this sentence at the end of your statement: *The funders had no role in study design, data collection and analysis, decision to publish, or preparation of the manuscript.*<br>• **YES** - Specify the role(s) played.<br><br>* typeset	This research project is supported by the National Research Council of Thailand (NCRT): NRCT-RGJ63003-068. The Thailand science research and innovation fund and the University of Phayao (Grant No. FF66-UoE013).
**Competing Interests**<br><br>Use the instructions below to enter a competing interest statement for this submission. On behalf of all authors, disclose any competing interests that could be perceived to bias this work—acknowledging all financial support and any other relevant financial or non-financial competing interests.	The authors have declared that no competing interests exist.

Powered by Editorial Manager® and ProduXion Manager® from Aries Systems Corporation

This statement is required for submission and **will appear in the published article** if the submission is accepted. Please make sure it is accurate and that any funding sources listed in your Funding Information later in the submission form are also declared in your Financial Disclosure statement.<br><br>View published research articles from *PLOS ONE* for specific examples.<br><br>**NO authors have competing interests**<br><br>Enter: *The authors have declared that no competing interests exist.*<br><br>**Authors with competing interests**<br><br>Enter competing interest details beginning with this statement:<br><br>*I have read the journal's policy and the authors of this manuscript have the following competing interests: [insert competing interests here]*<br><br>* typeset	
**Ethics Statement**<br><br>Enter an ethics statement for this submission. This statement is required if the study involved:<br><br>• Human participants<br>• Human specimens or tissue<br>• Vertebrate animals or cephalopods<br>• Vertebrate embryos or tissues<br>• Field research<br><br>Write "N/A" if the submission does not require an ethics statement.<br><br>General guidance is provided below. Consult the submission guidelines for detailed instructions. **Make sure that all information entered here is included in the Methods section of the manuscript.**	All procedures followed the authorization of the Animal Ethics Committee of KhonKaen University based on the Ethics of Animal Experimentation of the National Research Council of Thailand (Record No. IACUC-KKU-105/63), the animal care and use license for scientific research was U1-07864-2561 (COA.No.MU-IACUC 2019/007) for the fishes of the Mae Klong Basin in Thailand approved from Mahidol University and fish collection authorized by Department of fisheries in Thailand, license number was 49/2564, respectively.

Powered by Editorial Manager® and ProduXion Manager® from Aries Systems Corporation

**Format for specific study types**<br><br>**Human Subject Research (involving human participants and/or tissue)**<br>• Give the name of the institutional review board or ethics committee that approved the study<br>• Include the approval number and/or a statement indicating approval of this research<br>• Indicate the form of consent obtained (written/oral) or the reason that consent was not obtained (e.g. the data were analyzed anonymously)<br><br>**Animal Research (involving vertebrate animals, embryos or tissues)**<br>• Provide the name of the Institutional Animal Care and Use Committee (IACUC) or other relevant ethics board that reviewed the study protocol, and indicate whether they approved this research or granted a formal waiver of ethical approval<br>• Include an approval number if one was obtained<br>• If the study involved *non-human primates*, add *additional details* about animal welfare and steps taken to ameliorate suffering<br>• If anesthesia, euthanasia, or any kind of animal sacrifice is part of the study, include briefly which substances and/or methods were applied<br><br>**Field Research**<br><br>Include the following details if this study involves the collection of plant, animal, or other materials from a natural setting:<br>• Field permit number<br>• Name of the institution or relevant body that granted permission	
**Data Availability**<br><br>Authors are required to make all data underlying the findings described fully available, without restriction, and from the time of publication. PLOS allows rare exceptions to address legal and ethical concerns. See the PLOS Data Policy and FAQ for detailed information.	Yes - all data are fully available without restriction

Powered by Editorial Manager® and ProduXion Manager® from Aries Systems Corporation

A Data Availability Statement describing where the data can be found is required at submission. Your answers to this question constitute the Data Availability Statement and **will be published in the article**, if accepted.<br><br>**Important:** Stating 'data available on request from the author' is not sufficient. If your data are only available upon request, select 'No' for the first question and explain your exceptional situation in the text box.<br><br>Do the authors confirm that all data underlying the findings described in their manuscript are fully available without restriction?	
**Describe where the data may be found in full sentences. If you are copying our sample text, replace any instances of XXX with the appropriate details.**<br><br>• If the data are **held or will be held in a public repository**, include URLs, accession numbers or DOIs. If this information will only be available after acceptance, indicate this by ticking the box below. For example: *All XXX files are available from the XXX database (accession number(s) XXX, XXX.*).<br>• If the data are all contained **within the manuscript and/or Supporting Information files**, enter the following: *All relevant data are within the manuscript and its Supporting Information files.*<br>• If neither of these applies but you are able to provide **details of access elsewhere**, with or without limitations, please do so. For example:<br><br>*Data cannot be shared publicly because of [XXX]. Data are available from the XXX Institutional Data Access / Ethics Committee (contact via XXX) for researchers who meet the criteria for access to confidential data.*<br><br>*The data underlying the results presented in the study are available*	All relevant data are within the manuscript.

from (include the name of the third party and contact information or URL).<br>• This text is appropriate if the data are owned by a third party and authors do not have permission to share the data.<br><br>* typeset	
Additional data availability information:	

Powered by Editorial Manager® and ProduXion Manager® from Aries Systems Corporation

Manuscript
Click here to access/download;Manuscript;Manuscript.docx

Chromosomes of Asian cyprinid fishes: Novel insight into the chromosomal evolution of Labeoninae (Teleostei, Cyprinidae)

Sudarat Khensuwan[1☯]; Francisco de Menezes Cavalcante Sassi[2☯]; Renata Luiza Rosa de Moraes[2]; Petr Rab[3]; Thomas Liehr[4]; Weerayuth Supiwong[5*]; Kriengkrai Seetapan[6]; Alongklod Tanomtong[1]; Nathpapat Tantisuwichwong[1]; Satit Arunsang[7]; Phichaya Buasriyot[8]; Sampun Tongnunui[9]; Marcelo de Bello Cioffi[2]

[1]Department of Biology, Faculty of Science, Khon Kaen University, Muang, Khon Kaen 40002, Thailand; (SK) sudarat_k@kkumail.com, 0009-0007-4019-5489; (AT) tanomtong@hotmail.com, 0000-0002-8466-3594; (NT) naptan@kku.ac.th, 0000-0003-2423-6846

[2]Laboratório de Citogenética de Peixes, Departamento de Genética e Evolução, Universidade Federal de São Carlos, Rod. Washington Luiz, S/N, Jardim Guanabara, São Carlos, SP, 13565-905, Brazil. (FMCS) francisco.sassi@hotmail.com, 0000-0001-5699-5789; (RLRM) rlrdm@hotmail.com, 0000-0003-0969-8014; (MBC) mbcioffi@ufscar.br, 0000-0003-4340-1464

[3]Laboratory of Fish Genetics, Institute of Animal Physiology and Genetics, Czech Academy of Sciences, Rumburská, 89, Liběchov, 277 21, Czech Republic. (PR) rab@iapg.cas.cz, 0000-0002-1830-3523

[4]Institute of Human Genetics, University Hospital Jena, Jena 07747, Germany. (TL) thomas.liehr@med.uni-jena.de, 0000-0003-1672-3054

[5]Faculty of Applied Science and Engineering, Khon Kaen University, Nong Khai Campus, Muang,Nong Khai 43000, Thailand; (WS) weersu@kku.ac.th, 0000-0002-1670-3224

[6]School of Agriculture and Natural Resources, University of Phayao, Tumbol Maeka, Muang, Phayao 56000, Thailand; (KS) kook82@hotmail.com, 0000-0003-0900-6890

[7]Program in Animal Science, Faculty of Agricultural Technology and Agro-industry, Rajamangala University of Technology Suvarnabhumi, *Phra Nakhon* Si Ayutthaya, Ayutthaya, Thailand 13000; (SA) sathit.a@rmutsb.ac.th, 0009-0005-7574-5757

[8]Faculty of Science and Technology, Rajamangala University of Technology Suvarnabhumi, Mueang Nonthaburi, Nonthaburi Thailand, 11000; (PB) phichaya.b@rmutsb.ac.th, 0000-0003-0821-7629

[9]Department of Conservation Biology, Mahidol University, Kanchanburi Campus, Sai Yok, Kanchanaburi Province, 71150, Thailand; (ST) sampan.ton@mahidol.ac.th, 0009-0002-4899-908X

☯These authors contributed equally to this work.
*Corresponding author: (WS) weersu@kku.ac.th, 0000-0002-1670-3224

Abstract

The Labeoninae subfamily is a highly diversified but demonstrably monophyletic lineage of cyprinid fishes comprising five tribes and six incertae sedis genera. This widely distributed assemblage contains some 48 genera and around 480 recognized species distributed in freshwaters of Africa and Asia. In this study, the karyotypes and other chromosomal properties of five Labeoninae species found in Thailand *Labeo chrysophekadion* (Labeonini) and *Epalzeorhynchos bicolor*, *E. munense*, *Henicorhynchus siamensis*, *Thynnichthys thynnoides* (´Osteochilini´) were examined using conventional and molecular cytogenetic protocols. Our results confirmed a diploid chromosome number (2n) invariably 2n = 50, but the ratio of uni- and bi-armed chromosomes was highly variable among their karyotypes, indicating extensive structural chromosomal rearrangements. Karyotype of *L. chrysophekadion* contained 10m+6sm+20st+14a, 32m+10sm+8st for *H. siamensis*, 20m+12sm+10st+8a in *E. bicolor*, 20m+8sm+8st+14a in *E. munense*, and 18m+24sm+8st in *T. thynnoides*. Except for *H. siamensis*, which had four sites of 5S rDNA sites, other species under study had only one chromosome pair with those sites. In contrast, only one pair containing 18S rDNA sites were found in the karyotypes of three species, whereas two sites were found in that of *E. bicolor*. These cytogenetic patterns indicated that the cytogenomic divergence patterns of these labeonine species largely corresponded to the inferred phylogenetic tree. In spite the

2n stability, diverse patterns of rDNA and microsatellite distribution as well as their various karyotype structures demonstrated significant evolutionary differentiation of Labeoninae genomes as exemplified in examined species.

Keywords: Fish cytogenetics, karyotype evolution and stasis, FISH, repetitive DNAs

Introduction

Around 48 genera and more than 480 species make up the demonstrably monophyletic Labeoninae (Cyprinidae, Cypriniformes) [1], which inhabits the swift-moving rivers and streams of Africa and Asia [2]. These fishes are frequently objects of the aquarium trade due to their beautiful body patterns as well as artisanal fisheries, corresponding to 18% of all production of aquatic animals in 2020 [3]. Nonetheless, most cytogenetic research is limited to the report of the diploid chromosome number (2n) and the karyotype composition [4, 5]. All species of the subfamily Labeoninae are evolutionary diploid, with 2n ranging from 48 to 50, in contrast to the tetra- and hexaploid lineages of cyprinid subfamilies, namely Barbinae, Cyprininae, Probarbinae, Schizopygopsinae, Schizothoracinae, Smiliogastrinae, Spinibarbinae, and Torinae [4, 6, 7, 1]. Phylogenetic reconstructions suggest that the divergence of cyprinid lineages began with diploid species 70 Mya, progressed through a tetraploidization stage between 45 and 20 mya, and then experienced a more recent hexaploidization events in the last 20 mya. However, the exact timing of this divergence is still unknown [7]. According to Yang et al. [7], the Labeoninae family underwent diversification 43.6 million years ago and was found to be the sister basal branch of all cyprinids. [8, 6, 9, 7].

Representatives of evolutionary diploid subfamilies, Acrossocheilinae and ´Poropuntiinae´ both have a conserved 2n of 50, but those of Labeoninae and Smiliogastrinae vary between values 48 and 50 **(Table 1).** This reduction in 2n was caused by chromosomal fusions, which, in certain rare cases, are associated with origin of the sex chromosomes [10]. Despite the widespread conservation of their 2n, the Labeoninae were found to have differences in the macro- and microstructure of the karyotypes as evidenced by the different fundamental numbers (NF) and 18S rDNA sites distribution (Table 1) in species and populations, which suggests a significant role for pericentric inversions in the karyotype evolution. In this context, molecular cytogenetics emerges as a crucial tool to identify the karyotype dynamics also in fishes, as has already been demonstrated for several Cyprinidae species [11-18]. The mapping of repetitive sequences, which make up the majority of the eukaryotic genome, is widely utilized in fish cytogenetics and has a significant potential to further understanding the process of sex and B chromosomes as well as karyotype differentiation [19].

The use of molecular and conventional cytogenetic methods provides important data for the study of cyprinoid evolution. In general, available data demonstrate that although almost all cyprinoid species share the same macrostructure of their karyotypes (i. e: 2n=50 and karyotypes dominated by bi-armed chromosomes), they diverge in patterns of C-positive heterochromatin, and rDNA distribution [18, 20-26]. Hence, we provide here novel cytogenetic data for five Labeoninae species, namely *Epalzeorhynchos bicolor* Smith (1931), *E. munense* Smith (1934), *Henicorhynchus siamensis* Sauvage (1881), *Thynnichthys thynnoides* Bleeker (1852) and *Labeo chrysophekadion* Bleeker (1849) from two tribes (Osteochilini and Labeonini *sensu* Tan & Armbruster, 2018).

Table 1. Review of cytogenetic data in the tribe Labeoninae, excluding *incertae sedis* genera (sensu Tan and Armbruster [1]). The species analyzed in this study are highlighted in red.

Species	2n	NF	Karyotype	NORs/18S rDNA pairs	References
Bangana devdevi	50	86	20m+16sm+14a	-	[27]
Barbichthys laevis	50	76	20m+6sm+14a	-	[28]
Cirrhinus julleini	50	90	26m+14sm+4st+6a	-	[29]
Cirrhinus microlepis	50	72	12m+10sm+2st+26a	-	[30]
Cirrhinus molitorella	50	98	20m+26sm+2st+2a	-	[31]
Cirrhinus molitorella	50	100	16m+24sm+10st	four pairs	[32-35]
Cirrhinus mrigala	50	82	10m+12sm+10st+18a	two pairs	[29]
Cirrhinus mrigala	50	100	4m+18sm+28st	-	[36]
Cirrhinus mrigala	50	78	8m+6sm+14st+22a	-	[37]
Cirrhinus mrigala	50	86	12m+18sm+6st+14a	two pairs	[38]
Cirrhinus reba	50	78	6m+8sm+14st+22a	-	[37]
Cirrhinus reba	50	92	18m+20sm+6st+4a	-	[39]
Crossocheilus latius latius	50	90	12m+28sm+10a	-	[40, 41]
Crossocheilus latius latius	50	82	8m+12sm+12st+18a	-	[42]
Crossocheilus latius punjabensis	48	60	12m+36a	-	[43]
Discogobio tetrabarbatus	50	90	10m+18sm+12st+10a	-	[32,33,35]
Epalzeorhynchos bicolor	50	52	20m+4sm+2st+24a	-	[44]
Epalzeorhynchos bicolor	50	84	20m+12sm+10st+8a	19, 21	Present study
Epalzeorhynchos frenatum	48	72	14m+10sm+8st+16a	-	[44]
Epalzeorhynchos frenatum	50	76	18m+10sm+10st+12a	10	[5]
Epalzeorhynchos frenatum	50	72	20m+8sm+8st+14a	11	Present study
Epalzeorhynchos munensis	50	84	22m+12sm+2st+14a	-	[45]
Garra cambodgiensis	50	82	20m+12sm+4st+14a	-	[46]
Garra cambodgiensis	50	76	8m+18sm/st+24a	-	[47, 48]
Garra dembeensis	50	82	14m+18sm+18a	-	[49]
Garra fasciacauda	50	84	18m+14sm+2st+16a	-	[46]
Garra gotyla gotyla	50	84	14m+10sm+10st+16a	-	[50]
Garra gotyla gotyla	50	90	14m+26sm+10a	-	[51]
Garra gotyla gotyla	50	84	12m+8sm+8st+22a	-	[52]
Garra imberba	50	96	14m+20sm+12st+4a	-	[53, 35]
Garra kempi	50	88	14m+14sm+10st+12a	-	[52]
Garra lamta	50	88	12m+24sm+2st+12a	-	[54]
Garra lamta	50	86	6m+18sm+12st+14a	-	[55]
Garra lissorhynchus	50	88	16m+16sm+6st+12a	-	[52]
Garra makiensis	50	84	14m+20sm+16a	-	[49]
Garra mullya	50	92	18m+14sm+10st+8a	two pairs	[56]
Garra notata	50	80	20m+10sm+20a	-	[46]
Garra orientalis	50	92	16m+12sm+14st+8a	-	[32, 33,35]
Garra ornata	50	-	-	-	[57]
Garra quadrimaculata	50	88	16m+22sm+12a	-	[49]
Garra rufa obtusa	44-52	-	-	-	[58]
Garra rufa rufa	44-52	-	-	-	[58]
Garra rufa	44	86	22m+20sm+2a	-	[52]
Garra surendranathanii	50	92	14m+20sm+8st+8a	-	[56]
Garra trewavasae	50	65	10m+3sm+2st+35a	-	[59]
Garra variabilis	74	-	-	-	[58]
Henicorhynchus siamensis	50	100	32m+10sm+8st	18	Present study
Labeo alluaudi	50	-	-	-	[57]
Labeo bata	50	80	18m+12sm+8st+12a	two pairs	[60]
Labeo bata	50	74	6m+18sm+16st+10a	-	[39]
Labeo behri	50	70	12m+8sm+2st+28a	-	[61]

Labeo calbasu	50	70	10m+10sm+14st+16a	two pairs	[60]
Labeo calbasu	50	64	6m+8sm+22st+14a	-	[39]
Labeo calbasu	50	80	8m+22sm/st+20a	two pairs	[62, 63]
Labeo caeruleus	48	66	12m+6sm+6st+24a	-	[37]
Labeo chrysophekadion	50	78	4m+10sm+14st+22a	-	[64]
Labeo chrysophekadion	50	72	10m+6sm+20st+14a	12,13	[65]
Labeo chrysophekadion	50	72	10m+6sm+20st+14a	9	Present study
Labeo coubie	50	-	-	-	[66]
Labeo dero	50	88	26m+12sm+2st+10a	-	[67]
Labeo dero	48	76	12m+16sm+20st/a	-	[68]
Labeo diplostomus	50	66	10m+6sm+8st+26a	-	[50]
Labeo dussumieri	50	74	12m+12sm+10st+16a	four pairs	[69]
Labeo gonius	54	54	54a	two pairs	[43]
Labeo pangusia	50	68	6m+12sm+16st+16a	-	[42]
Labeo parvus	50	-	-	-	[57]
Labeo parvus	50	-	-	-	[57]
Labeo rohita	50	80	14m+16sm+8st+12a	two pairs	[29]
Labeo rohita	50	74	10m+14sm+6st+20a	two pairs	[60]
Labeo rohita	50	80	10m+20sm+20a	-	[36]
Labeo rohita	50	70	8m+12sm+6st+24a	-	[70]
Labeo rohita	50	74	10m+14sm+8st+18a	four pairs	[38]
Labeo rohita	50	88	10m+16sm+12st+12a	-	[32, 33, 35]
Labeo roseopunctatus	50	-	-	-	[66]
Labeo rouaneti	50	-	-	-	[57]
Labeo senegalensis	50	-	-	-	[66]
Labiobarbus lineatus	50	80	20m+10st+20a	-	[71]
Labiobarbus leptocheilus	50	86	14m+6sm+16st+14a	2	[17]
Lobocheilos rhabdoura	50	96	10m+18sm+12st+10a	6	[26]
Osteochilus hasselti	50	96	30m+14sm+6st	-	[71]
Osteochilus lini	50	100	12m+34sm+4st	12	[18]
Osteochilus melanopleura	50	96	22m+24sm+2st+2a	2	[18]
Osteochilus microcephalus	50	100	14m+32sm+4st	3	[18]
Osteochilus vittatus	50	96	16m+30sm+4st	12	[18, 71]
Osteochilus waandersi	50	92	18m+24sm+4st+4a	15	[29, 18]
Parasinilabeo assimilis	50	96	16m+12sm+18st+4a	-	[32, 35]
Semilabeo notabilis	50	80	8m+10sm+12st+20a	-	[32, 35]
Semilabeo prochilus	50	98	16m+18sm+14st+2a	-	[35, 53]
Semilabeo prochilus	50	100	16m+20sm+14st	seven pairs	[72]
Thynnichthys thynnoides	50	100	18m+24sm+8st	10	Present study

Material and Methods

Sampling

As shown in Figure 1 and Table 2, samples ~~have been~~ collected in five different locations, that belong to Thailand's Mae Klong, Songkhram, and Chao Phraya drainage systems. ~~We included~~ ~~two species that~~ were

collected in the same basins~~, these two species~~ ~~were~~ very low abundance in wild habitats [73, 74] as well as bloodstock wilds ~~have been~~ successfully ~~artificial~~ ~~insemination~~ in the aquaculture farms in Thailand. Hence, fish samples were purchased directly from the Ban Pong Fish Market in Ratchaburi province. The analysed individuals ~~have been~~ deposited in the fish collections at KhonKaen University's Cytogenetic Laboratory, Department of Biology, and Faculty of Science.~~,~~ All the species examined ~~here~~ were correctly identified using the morphological criteria [75, 76]. Mitotic chromosomes were obtained from the anterior kidney according to Bertollo et al. [77]. We used silver nitrate ($AgNO_3$) to visualize the nucleolar organizer regions (Ag-NOR) [78]. All procedures followed the authorization of the Animal Ethics Committee of KhonKaen University based on the Ethics of Animal Experimentation of the National Research Council of Thailand (Record No. IACUC-KKU-105/63), the animal care and use license for scientific research was U1-07864-2561 (COA.No.MU-IACUC 2019/007) for the fishes of the Mae Klong Basin in Thailand approved from Mahidol University and fish collection authorized by Department of fisheries in Thailand, license number was 49/2564, respectively.

~~**Table 2**. The collection sites of the five Labeoninae species and the number of analyzed individuals (n) per sex. Individuals of *E. bicolor* and *E. munense* were gathered from Ban Pong Fish Market, Ratchaburi province (13°51'42.6"N 99°52'48.8"E).~~

~~**Figure 1.** Thailand map showing the collection sites of the five species studied including 1. *Labeo chrysophekadion* (green circles); 2. *Henicorhynchus siamensis* (orange circles); 3. *Epalzeorhynchos bicolor* (pink circles); 4. *Epalzeorhynchos munense* (yellow circles); 5. *Thynnichthys thynnoides* (red circles). Scale bar = 1 cm.~~

Fluorescence in situ hybridization (FISH)

Fluorescence *in situ* hybridization experiments were conducted under high stringency conditions [79] to identify both classes of ribosomal DNA (5S and 18S) and microsatellites $(CA)_{15}$, $(GC)_{15}$, $(TA)_{15}$, $(CAA)_{10}$ $(GAA)_{10}$ $(CAG)_{10}$ $(CAT)_{10}$ and $(CGG)_{10}$ sequences. The former ribosomal probe contained 120 base pairs (bp) of the 5S rRNA transcribing gene in addition to 200 bp of the non-transcribed spacer (NTS) [80]. The later one corresponded to the 1400 bp segment of the 18S rRNA gene obtained *via* PCR from the nuclear DNA of *Hoplias malabaricus* [81]. Both rDNA probes were directly labelled with the Nick-Translation mix kit (Jena Bioscience, Jena, Germany), where 5S rDNA was labeled in red with Atto550-dUTP and the 18S rDNA was labelled in green with Atto448-dUTP, ~~according to the manufacturer's instructions~~. The microsatellite sequences were directly labelled with Cy-3 during the synthesis~~, described~~ by Kubat et al. [82].

Image processing

To confirm the 2n, karyotype structure, and FISH results, we used at least 20 metaphase spreads per individual. Images were captured with an Axioplan II microscope (Carl Zeiss Jena GmbH, Germany) with CoolSNAP, and processed using Image-Pro Plus 4.1 software (Media Cybernetics, Silver Spring, MD, USA). Chromosomes were classified as metacentric (m), submetacentric (sm), subtelocentric (st), and acrocentric (a) ~~according to~~ Levan et al. [83].

Results

All five examined species, for both females and males, possessed invariably, 2n = 50, but with distinct karyotype compositions: 10m+6sm+20st+14a in *Labeo chrysophekadion*, 32m+10sm+8st in *Henicorhynchos*

siamensis **(Figure 2)**, 20m+12sm+10st+8a in *Epalzeorhynchos bicolor*, 20m+8sm+8st+14a in *E. munense*, and 18m+24sm+8st in *Thynnichthys thynnoides* **(Figure 3)**. Single Ag-NOR site were detected in all species at the telomeric region of p arms. In the genome of *E. bicolor* and *E. munense*, the 5S rDNA was distributed in the centromeric region of st pairs 20 and 15. On the other hand, *H. siamensis* had these sites located in four chromosomal pairs at the centromeric regio[illegible]irs 3, 6, 17, 19). For *L. chrysophekadion* (pair 7, **Figure 2**) and *T. thynnoides* (pairs 17, 22, **Figure 3**), the 5S rDNA was found in [illegible] telomeric region of the p arms. The 18S rDNA distribution corresponded to the Ag-NOR signals: pair 9 in *L. chrysophekadion*, pair 18 in *H. siamensis* pairs 19 and 21 in *E. bicolor*, pair 11 in *E. munense*, and pair 10 in *T. thynnoides*.

Figure 2. Karyotypes of *Labeo chrysophekadion* (A,C,E) and *Henicorhyncus siamensis* (B,D,F) organized from chromosomes after Giemsa-staining, Ag-NOR impregnation and FISH with 5S and 18S probes. Chromosomes were counterstained with DAPI (blue). Bar = 5 µm.

Figure 3. Karyotypes of *Epalzeorhyncos bicolor* (A,C,E), *E. munense* (B,D,F), and *Thynnichthys thynnoides* (G,H,I) arranged from Giemsa- and Ag-NOR-stained chromosomes and chromosomes after FISH with 5S (red) and 18S (green) rDNA probes. Chromosomes were counterstained with DAPI (blue). Scale bar = 5 µm.

Microsatellite mapping showed species-specific patterns and mostly telomeric signals for all species **(Figures 4-8)**. For the (CA)$_{15}$, dispersed signals were seen in the chromosomes of *L. chrysophekadion*, whereas *T. thynnoides* and *H. siamensis* only displayed telomeric signals, in contrast to *E. bicolor* and *E. munense*, which additionally display one set of signals marked in the pericentromeric region. The (GC)$_{15}$ was found in the telomeres of *T. thynnoides* and *E. munense*; however, in *E. bicolor* and *H. siamensis*, only one chromosome pair was marked, and this motif was dispersed in *L. chrysophekadion*. The (GAA)$_{10}$ accumulates in the telomeres of other species but was distributed in the chromosomes of *T. thynnoides* and *E. munense*. On the other hand, (CAT)$_{10}$ was only accumulated in telomeres of *E. bicolor*, in contrast to dispersed in other species. All species, except *E. munense* with a single chromosome pair marked, had telomeric signals for the (CAA)$_{10}$ probe. The (TA)$_{15}$ was dispersed in *L. chrysophekadion* and *H. siamensis* chromosomes, but accumulated in telomeres of all other species. The (CAG)$_{10}$ was found dispersed in *L. chrysophekadion* and *T. thynnoides*, in the telomeres of all chromosomes of *E. munense*, restricted to the telomeric region of a single chromosome pair in *E. bicolor*, and bitelomeric in a single pair of *H. siamensis* in addition to a telomeric mark on short arm of a metacentric pair. Finally, in *H. siamensis* and *T. thynnoides*, the (CGG)$_{10}$ probe was found in the telomeres of all chromosomes, but in *E. munense*, it was only identified as a strong signal on one pair in the centromeric region, in contrast to a scattered pattern in *E. bicolor* and *T. thynnoides* chromosomes.

Figure 4. Distribution of microsatellite sequences (CA)$_{15}$, (GC)$_{15}$, (TA)$_{15}$, (CAA)$_{10}$ (GAA)$_{10}$ (CAG)$_{10}$ (CAT)$_{10}$ and (CGG)$_{10}$ onto *L. chrysophekadion* metaphase chromosomes.

Figure 5. Distribution of microsatellite sequences (CA)$_{15}$, (GC)$_{15}$, (TA)$_{15}$, (CAA)$_{10}$ (GAA)$_{10}$ (CAG)$_{10}$ (CAT)$_{10}$ and (CGG)$_{10}$ onto *H. siamensis* metaphase chromosomes.

Figure 6. Distribution of microsatellite sequences (CA)$_{15}$, (GC)$_{15}$, (TA)$_{15}$, (CAA)$_{10}$ (GAA)$_{10}$ (CAG)$_{10}$ (CAT)$_{10}$ and (CGG)$_{10}$ onto *E. bicolor* metaphase chromosomes.

Figure 7. Distribution of microsatellite sequences $(CA)_{15}$, $(GC)_{15}$, $(TA)_{15}$, $(CAA)_{10}$ $(GAA)_{10}$ $(CAG)_{10}$ $(CAT)_{10}$ and $(CGG)_{10}$ onto *E. munense* metaphase chromosomes.

Figure 8. Distribution of microsatellite sequences $(CA)_{15}$, $(GC)_{15}$, $(TA)_{15}$, $(CAA)_{10}$ $(GAA)_{10}$ $(CAG)_{10}$ $(CAT)_{10}$ and $(CGG)_{10}$ onto *T. thynnoides* metaphase chromosomes.

Discussion

For the great majority of species reviewed in Table 1, including those in this study, Labeoninae has 2n = 50, which is also hypothesized as a basal for cypriniform fishes [18, 84, 85]. Even within *Epalzeorhynchos* species, considerable variations in karyotype structures were observed in both species as well as in other ones herein investigated species, despite their conservative 2n. The different proportions of bi-armed chromosomes (metacentric and submetacentric) in the karyotypes imply that rearrangements such as pericentric inversions may have acted during their karyotypic differentiation. It is also remarkable that a large variety of karyotype re-organization occurs among populations, so as in the distribution of Ag-NOR/18S rDNA. Despite the fact that rDNAs are conservative elements of the eukaryotic genomes [86], the 18S rDNA typically occupies a terminal position in chromosomes, suggesting that these regions could represent recombination hotspots [87]. Also, in ´Poropuntiinae´ (Cyprinidae) 5S rDNA shows a centromeric distribution as observed in *Hypsibarbus* spp. and *Mystacoleucus* spp. [88]. Ind[illegible] rDNA elements showed remarkable different patterns among cyprinid species, especially regarding the variability in the number and position of the 5S rDNA sites compared to the more stable pattern of the 18S rDNA sites. Such differences were notable in the sister c[illegible]s *Epalzeorhynchos* and *Henichorhynchus*, where the former has a single chromosome pair carrying the 5S rDNA and the last four pairs. The mapping of repetitive sequences, especially ribosomal genes, has proven useful for estim[illegible]g evolutionary karyotype changes [87].

We demonstrated that the karyotype of individuals herein investigated of *E. bicolor* is notably distinct from that one described by Donsakul and Magtoon [89] (treated as *Labeo bicolor*). In their report, the karyotype contains a higher number of a chromosomes (24) than in our study (8). Such an enormous difference does not occur in description of the karyotype of *E. munense*, where the acrocentrics vary from 6 to 8 pairs among populations. The extensive numerical variation of acrocentric chromosomes indicates that pericentric inversions might occurred during the differentiation of their karyotypes. These rearrangements have been hypothesized to be crucial for local speciation and adaptation as well as for recombination suppression, especially in sex chromosomes [90-95]. This anticipated mechanism is widespread in cyprinoids chromosome evolution, since most maintain the common 2n = 50, but variable in the karyotype composition. Both *Epalzeorhynchos* species also greatly differ in the distri[illegible]n of microsatelite sequences, as for the $(GC)_{15}$, $(CAA)_{10}$ and $(CAG)_{10}$ microsatellites. Although mostly restricted to telomeric regions, where a significant fraction of repetitive DNA is localized [19], the number of chromosomes carrying these abovementioned motifs suggests that the dynamics of repetitive DNAs can be related to species divergences of this genus. In [illegible] genome evolution of various fish groups, the role of repetitive DNAs has been demonstrated [17-20, 96-100,].

The proportion of biarmed chromosomes in Cyprinidae karyotypes is typically significantly greater than that of uniarmed ones [85]. The *Epalzeorhynchos* + *Henicorhynchus* + *Thynnichthys* clade (Osteochilini) has a significantly higher number of (20) metacentric chromosomes than its sister clade Labeonini, which possessed approximately 10 metacentric chromosomes making up their karyotypes as exemplified here in *L.*

chrysophekadion (Figure 10). However, *Labeo bata* and *L. dero* have a more similar pattern to Osteochilini, with 18 and 26 metacentrics in karyotypes, respectively. The same higher proportion of metacentrics also occurs in Garrini [4] and a high number of ~~m~~ chromosomes might be a plesiomorphic feature of the Osteochilini and Garrini tribes, while the lower metacentric number in Labeonini could represent an derived feature of this tribe.

~~**Figure 9**. Mapping of our cytogenetic results onto modified phylogenetic relationships of the Labeoninae based on nuclear and mitochondrial genes by Yang et al. [2] and classification of Tan and Armbruster [1]. The common names of the species are indicated under the scientific name. The position of *Epalzeorhynchos munense* was manually added to our figure, since it is not included in the [2] phylogeny, thus requiring confirmation.~~

Numerous fish species have the ability to conserve 2n value. Karyotype stasis/conservatism is primarily observed in marine fishes and is associated with a high degree of synteny between various DNA classes (e.g. Ellegren [101], Zhang et al. [102]). However, the conservation of 2n frequently follows the substantial changes in the karyotype structures and the distribution of repetitive DNAs in some families of Cyprinoidea. For example, the great 2n variation spanning from 42 in *Acheilognathus gracilis* (Acheilognathidae) [103] to 446 in *Ptychobarbus dipogon* (Cyprinidae) [104] has been recorded for ~~by~~ families that experienced polyploidy throughout their chromosome evolution processes. In this context, the maintenance of 2n = 50 could represent the conservation of an ancestral trait in cyprinids [84, 85].

~~Our findings~~ contributed to the knowledge of karyotypes and chromosome characteristics in the Labeoninae. Overall, these characteristics show that structural chromosome rearrangements, such as pericentric inversions, ~~were~~ mainly in ~~charge~~ of the wide numerical diversity of acrocentric chromosomes and ~~had~~ a major impact on the evolution of this cyprinid group. According to the detailed cytogenetic survey, trends of cytogenomic divergences can be identified in these Labeoninae species, which largely correspond to the inferred phylogenetic tree. Repetitive DNAs that demonstrated specificity in their distribution among species, such as ribosomal and microsatellite DNAs, ~~were~~ also effective indicators and drivers of particular genomic differentiation within Labeoninae.

Acknowledgements: This research project is supported by the National Research Council of Thailand (NCRT): NRCT-RGJ63003-068. The Thailand science research and innovation fund and the University of Phayao (Grant No. FF66-UoE013).

References

1. Tan M, Armbruster JW. Phylogenetic classification of extant genera of fishes of the order Cypriniformes (Teleostei: Ostariophysi). Zootaxa 2018;4476(1): 6-39. doi: 10.11646/zootaxa.4476.1.4
2. Yang L, Arunachalam M, Sado T, Levin BA, Golubtsov AS, Freyhof J, Friel JP, Chen W, Hirt MV, Manickam R, Agnew MK, Simons AM, Saitoh K, Miya M, Mayden RL, He S. Molecular phylogeny of the cyprinid tribe Labeonini (Teleostei: Cypriniformes). Mol. Phylogenet. Evol. 2012;65(2): 362-379. doi: 10.1016/j.ympev.2012.06.007
3. FAO. The State of World Fisheries and Aquaculture. 2022. Towards Blue Transformation: Rome; 2022. doi: 10.4060/cc0461en. https://www.fao.org/3/cc0461en/cc0461en.pdf
4. Arai R. Fish karyotypes: a check list. 1st ed.Springer Science & Business Media; 2011.
5. Phimphan S, Chaiyasan P, Suwannapoom C, Reungsing M, Juntaree S, Tanomtong A, Supiwong W. Comparative karyotype study of three Cyprinids (Cyprinidae, Cyprininae) in Thailand by classical

cytogenetic and FISH techniques. Comp. Cytogenet. 2020;14(4):597. doi: 10.3897/CompCytogen.v14i4.54428

6. Yang L, Sado T, Hirt MV, Pasco-Viel E, Arunachalam M, Li J, Wang X, Freyhof J, Saitoh K, Simons AM, Miya M, He S, Mayden RL. Phylogeny and polyploidy: resolving the classification of cyprinine fishes (Teleostei: Cypriniformes). Mol. Phylogenet. Evol. 2015;85: 97-116. doi: 10.1016/j.ympev.2015.01.014
7. Yang L, Naylor GJ, Mayden RL. Deciphering reticulate evolution of the largest group of polyploid vertebrates, the subfamily cyprininae (Teleostei: Cypriniformes). Mol. Phylogenet. Evol. 2022;166: 107323. doi: 10.1016/j.ympev.2021.107323
8. Conway KW. Osteology of the South Asian Genus *Psilorhynchus* McClelland, 1839 (Teleostei: Ostariophysi: Psilorhynchidae), with investigation of its phylogenetic relationships within the order Cypriniformes. Zool. J. Lin. Soc. 2011;163: 50-154. doi: 10.1111/j.1096-3642.2011.00698.x
9. Stout CC, Tan M, Lemmon AR, Lemmon EM, Armbruster JW. Resolving Cypriniformes relationships using an anchored enrichment approach. BMC Evol. Biol. 2016;16: 1-13. doi: 10.1186/s12862-016-0819-5
10. Kim DS, Song HY, Bang IC, Nam YK. Cytogenetic analysis of Korean shinner, *Coreoleuciscus splendidus* (Cyprinidae). J. Aquac. 2007;20(2): 140-143.
11. Sola L, Gornung E. Classical and molecular cytogenetics of the zebrafish, *Danio rerio* (Cyprinidae, Cypriniformes): an overview. Genetica 2001;111: 397-412.
12. Rábová M, Ráb P, Ozouf-Costaz C, Ene C, Wanzeböck J. Comparative cytogenetics and chromosomal characteristics of ribosomal DNA in the fish genus *Vimba* (Cyprinidae). Genetica 2003;118: 83-91. doi: 10.1023/a:1022965302614.
13. Libertini A, Sola L, Rampin M, Rossi A.R., Iijima K, Ueda T. Classical and molecular cytogenetic characterization of allochthonous European bitterling *Rhodeus amarus* (Cyprinidae, Acheilognathinae) from Northern Italy. Genes Genet. Syst. 2008;83(5): 417-422. doi: 10.1266/ggs.83.417
14. Boron A, Porycka K, Ito D, Abe S, Kirtiklis L. Comparative molecular cytogenetic analysis of three *Leuciscus* species (Pisces, Cyprinidae) using chromosome banding and FISH with rDNA. Genetica 2009;135: 199-207. doi: 10.1007/s10709-008-9269-3
15. Bishani A, Prokopov DY, Romanenko SA, Druzhkova AS, Interesova EA, Graphodatsky AS, Trifonov VA. Repetitive DNA characterization in Cyprinid species with different ploidy level. In: Chromosomes and Mitosis (pp. 25-25). Russia: Novosibirsk National Research State University; 2019. pp. 25-25.
16. Aiumsumang S, Chaiyasan P, Khoomsab K, Supiwong W, Phim-phan, ATS. Comparative chromosome mapping of repetitive DNA in four min-now fishes (Cyprinidae, Cypriniformes). Caryologia 2022;75(2): 71-80. doi:10.36253/caryologia-1523
17. Saenjundaeng P, Kaewmad P, Supiwong W, Pinthong K, Pengseng P, Tanomtong A. Karyotype and characteristics of nucleolar organizer regions in longfin carp, *Labiobarbus leptocheilus* (Cypriniformes, Cyprinidae). Cytologia 2018;83(3): 265-269. doi:10.1508/cytologia.83.265
18. Saenjundaeng P, Supiwong W, Sassi FMC, Bertollo LAC, Rab P, Kretschmer R, Tanomtong A, Suwannapoom C, Reungsing M, Cioffi MB. Chromosomes of Asian cyprinid fishes: Variable karyotype patterns and evolutionary trends in the genus *Osteochilus* (Cyprinidae, Labeoninae,“Osteochilini”). Genet. Mol. Biol. 2020;43: e20200195. doi: 10.1590/1678-4685-GMB-2020-0195
19. Cioffi MB, Bertollo LAC. Chromosomal distribution and evolution of repetitive DNAs in fish. Genome

Dyn. 2012;7: 197-221. doi: 10.1159/000337950

20. Yüksel E, Gaffaroğlu M. The analysis of nucleolar organizer regions in *Chalcalburnus mossulensis* (Pisces: Cyprinidae). J. fish. Sci. 2008;2(4): 587-591. doi:10.3153/jfscom.2008021
21. Gromicho M, Ozouf-Costaz C, Collares-Pereira MJ. Lack of correspondence between CMA3-, Ag-positive signals and 28S rDNA loci in two Iberian minnows (Teleostei, Cyprinidae) evidenced by sequential banding. Cytogenet. Genome Res. 2005;109(4): 507-511. doi: 10.1159/000084211
22. Knytl M, Kalous L, Symonová R, Rylková K, Ráb P. Chromosome studies of European cyprinid fishes: cross-species painting reveals natural allotetraploid origin of a *Carassius* female with 206 chromosomes. Cytogenet. Genome Res. 2013;139(4): 276-283. doi: 10.1159/000350689
23. Kirtiklis L, Ocalewicz K, Wiechowska M, Boroń A, Hliwa P. Molecular cytogenetic study of the European bitterling *Rhodeus amarus* (Teleostei: Cyprinidae: Acheilognathinae). Genetica 2014;142: 141-148. doi: 10.1007/s10709-014-9761-x
24. Li S, Zhou Y, Yang C, Fan S, Huang L, Zhou T, Wang Q, Zhao R, Tang, Tao M, Liu S. Comparative analyses of hypothalamus transcriptomes reveal fertility-, growth-, and immune-related genes and signal pathways in different ploidy cyprinid fish. Genomics 2021;113(2): 595-605. doi: 10.1016/j.ygeno.2021.01.004
25. Gaffaroğlu M, Karasu-Ayata M, Unal-Karakus S. Karyomorphology of Two Cyprinid Barbels (Teleostei: Cyprinidae) from Gediz River, Turkey. Cytol. Genet. 2022;56: 41–547. doi: 10.3103/S0095452722060032
26. Kentachalee N, Pinthong K, Tongnunui S, Tanomtong A, Juntharat S, Supiwong W. First Report on Genetic Structure of *Lobocheilos rhabdoura* (Fowler, 1934) (Labeoninae, Cyprinidae) in Thailand: Insight the Basic Knowledge for Fish Breeding. FAST 2023;9: 90-103.
27. Donsakul T, Rangsiruji A, Magtoon W. Karyotypes of five cyprinid fishes (Family Cyprinidae): *Bangana devdevi*, *Osteobrama alfrediana*, *Systomus stoliczkanus*, *Oxygaster anomalura* and *Aspidoparia morar* in Thailand. In: Proceedings of the 49th Kasetsart University Annual Conference: Fisheries. Thailand: Kasetsart University; 2011. pp. 169-177
28. Donsakul T, Magtoon W, Rangsiruji A. Karyological studies of four Cyprinid fishes: *Barbichthys nitidus*, *Mystacoleucus argenteus*, *Cychocheilichthys lagleri* and *Systomus* sp. 1 from Thailand. In: Proceedings of the 44rd Kasetsart University Annual Conference: Fisheries. Thailand: Kasetsart University; 2006.
29. Magtoon W, Arai R. Karyotypes and distribution of nucleolus organizer regions in cyprinid fishes from Thailand. J. Ichthyol. 1993;40(1): 77-85.
30. Donsakul T, Rangsiruji A, Magtoon W. Karyotypes of four cyprinid fishes: *Poropuntius normani*, *Hypsibarbus malcolmi*, *Scaphognathops bandanensis* and *Henicorhynchus caudiguttatus* from Thailand. In: Proceedings of the 45th Kasetsart University Annual Conference: Fisheries. Thailand: Kasetsart University; 2007. pp. 740-748.
31. Zhang JX, Liu XF, Wang ZX, Jin GQ. A comparative study on the karyotypes among the hybrid fish (*Sinilabeo decorus tungting* male × *Cirrhinus molitorella* female) and its parental fishes. Acta Hydrobiol. Sin. 1984;8: 313-320.
32. Gui JF, Li YC, Li K, Zhou T. Studies on the karyotype of Chinese cyprinid fish. (VII) Karyotypic analyses of fifteen species of Barbinae with consideration for their phyletic evolution. Transactions Chi. Ichthyol. Soc. 1986;5: 119-127.
33. Ren XH, Yu XJ. Characterization of nucleolar organizer regions of twelve species of Chinese cyprinid

fishes. Caryologia 1993;46: 201-207. doi: 10.1080/00087114.1993.10797260

34. Ren XH, Cui J, Yu Q. Chromosomal heterochromatin differentiation of cyprinid fishes. Genetica 1992;87: 47-51.

35. Yu XJ, Zhou T, Li YC, Li K, Zhou M. Chromosomes of Chinese freshwater fishes. Science Publishing House: USA; 1989.

36. Lakra WS & Krishna G. G-banded karyotypes of three species of Indian major carps. CIS 1994;57: 23-25.

37. Rishi, K.K. Chromosomal studies on four cyprinid fishes. Int. J. Acad. Ich. 1981;2: 1-4.

38. Nagpure NS, Kushwaha B, Srivastava SK, Ponniah AG. Comparative cytogenetic studies in Indian major carps *Labeo rohita*, *Catla catla* and *Cirrhinus mrigala* (Cyprinidae, Pisces). Chromosome Sci. 2001;5: 153-158.

39. Manna GK, Khuda-Bukhsh AR. Karyomorphology of cyprinid fishes and cytological evaluation of the family. Nucleus 1977;20: 119-127.

40. Khuda-Bukhsh AR, Rahman A, Chanda T, Nayak K, Khuda-Bukhsh A. Diploid numbers and chromosome formulae of some 29 species of Indian teleosts (Pisces). CIS 1995;58: 38-39.

41. Chanda T. *Aspidoparia morar*, *Chagunius chagunio*, *Crossocheilus latius latius*, *Mystus bleekeri*, *Mystus menoda menoda*, *Ailia coila*, *Clupisoma garua*, *Pseudeutropius atherinoides*, *Glyptothorax trilineatus*, *Laguvia rebeiroi*, *Nangra punctata*, *Johnius dussumieri*, *Colisa sota*. In: Ponniah AG, John G, editors. Fish chromosome atlas. India: National Bureau of Fish Genetic Resources; 1998. pp. 22–23, 48–49, 58–59, 158–159, 164–165, 190–191, 192–193, 196–197, 208–213, 244–245, 286–287).

42. Tripathi NK, Sharma OP. Cytological studies on six cyprinid fishes. Genetica 1987;73: 243-246.

43. Lakra WS, Rishi KK. Chromosomes of Indian fishes: an annotated list. Indian J. Anim. Sci. 1991;61: 342-349.

44. Donsakul T, Magtoon W. A chromosome study of three Cyprinid fishes, *Labeo erythrurus*, *L. bicolor* and *L. rohita*. In: Proceedings of the 31st Kasetsart University Annual Conference. Thailand: Kasetsart University; 1993.

45. Donsakul T, Magtoon W, Rangsiruji A. Karyotypes of five cyprinid fishes (family Cyprinidae): *Macrochirichthys macrochirus*, *Scaphiodonichthys acanthopterus*, *Epalzeorhynchos munensis*, *Opsarius koratensis* and *Parachela* sp. from Thailand. In: Proceedings of the 50th Kasetsart University Annual Conference. Thailand: Kasetsart University; 2012. pp. 439-446.

46. Donsakul T, Runsiruji A, Magtoon W. Karyotypes of three species of cyprinid fishes *Garra cambodgiensis*, *G. fasciacauda* and *G. notata* (Cypriniformes, Cyprinidae) from Thailand. Knowl. Res. 2016;2(2): 30-35. doi: 10.14456/randk.2016.14

47. Suzuki A, Taki Y, Mochizuki M, Hirata J. Chromosomal speciation in Eurasian and Japanese Cyprinidae (Pisces, Cypriniformes). Cytobios 1995;83: 171-186.

48. Hinegardner R, Rosen DE. Cellular DNA content and the evolution of teleostean fishes. Am. Nat. 1972;106(951):621-644.

49. Krysanov EY, Golubtsov AS. Karyotypes of three Garra species from Ethiopia. J. Fish Biol. 1993;42(3):465-467.

50. Khuda-Bukhsh AR. Karyotype studies in two species of hill stream fishes, *Labeo diplostomus* and *Garra gotyla gotyla* (Pisces, Cyprinidae). Cytol. Genet. 1984;4:421-424.

51. Khuda-Bukhsh AR, Barat A. Chromosomes in fifteen species of Indian teleosts (Pisces). Caryologia 1987;40(1-

2):131-144.

52. Sahoo PK, Nanda P, Barat A. Karyotypic Analysis of *Neolissocheilus hexagonolepis* (McClelland), *Puntius ticto* (Ham.) and *P. chola* (Ham.) (Family: Cyprinidae, Pisces). Cytologia 2007;72(4):409-413. doi: 10.1508/cytologia.72.409
53. Li K, Li YC, Gui JF, Xie XZ, Zhou T. Studies on the karyotypes of Chinese cyprinid fishes. IX. Karyotypes of nine species of Abramidinae and one species of Xenocyprininae. Acta Hydrobiol. Sin. 1986;10:189-193.
54. Khuda-Bukhsh AR, Chanda T, Barat A. Karyomorphology and evolution in some Indian hillstream fishes with particular reference to polyploidy in some species. Indo-Pac. Fishes 1986; 886-898.
55. Khuda-Bukhsh AR, Gupta SK, Goswami S. Karyotypic studies in Garra lamta and Mystus cavassius (Pisces). J. Anim. Sci. 1980;89: 557-562.
56. Nagpure NS, Kumar R, Srivastava SK, Kushwaha B, Gopalakrishnan A, Basheer VS. Cytogenetic characterization of two marine ornamental fishes, Chaetodon collare and Stegastes insularis. J. Mar. Biol. Ass. 2006;48(2):267-269.
57. Guégan JF, Morand S. Polyploid hosts: strange attractors for parasites?. Oikos 1996;77(2):366-370. doi: 10.2307/3546079
58. Duran A, Barquinero JF, Caballín MR, Ribas M, Puig P, Egozcue J, Barrios L. Suitability of FISH painting techniques for the detection of partial-body irradiations for biological dosimetry. Radiat. Res. 2002; 157(4):461-468. doi: 10.1667/0033-7587(2002)157[0461:SOFPTF]2.0.CO;2
59. Olaoluwa PM, Idowu AI. Karyological Studies of Garra trewavasae Monod 1950 from Jos Plateau, Nigeria. Environtropica 2019;(15):120-128.
60. John G, Barat A, Lakra WS. 1993. Localization of nucleolar organizer regions in *Labeo* (Cyprinidae). La Kromosomo 1993;70:2381-2384.
61. Arai R, Magtoon W. Karyotypes of four cyprinid fishes from Thailand. Bull. Nat. Mus. Nat. Sci. 1991;17: 183-188.
62. Rishi KK, Rishi S. Giemsa banding in fish chromosomes. Cytol. Genet. 1981;3:103-106.
63. Rock J, Eldridge M, Champion A, Johnston P, Joss J. Karyotype and nuclear DNA content of the Australian lungfish, *Neoceratodus forsteri* (Ceratodidae: Dipnoi). Cytogenet. Cell Genet. 1996;73:187-189. doi: 10.1159/000134336
64. Seetapan K. Karyotypes of sex fish species of the family Cyprinidae. In: Proceedings of the 45th Kasetsart University Annual Conference: Fisheries. Thailand: Kasetsart University; 2007. pp. 749-758
65. Sărășan A, Józsa E, Ardelean AC, Drăguț L. Sensitivity of geomorphons to mapping specific landforms from a digital elevation model: A case study of drumlins. Area 2019;51(2):257-267. doi: 10.1111/area.12451
66. Paugy D, Guégan JF, Agnèse JF. Three simultaneous and independent approaches to the characterization of a new species of *Labeo* (Teleostei, Cyprinidae) from West Africa. Can. J. Zoo. 1990;68:1124-1131.
67. Khuda-Bukhsh AR, Chanda T. Somatic chromosomes of three species of hillstream fishes from Assam. In: Das P, Jhingran AG, editors. Fish genetics in India. Today & Tomorrow's Printers and Publishers; 1989. pp. 69-73.
68. Kligerman AD, Bloom SE. Rapid chromosome preparations from solid tissues of fishes. J. Fish. Res. Board Can. 1977;34:266-269.
69. Nagpure NS, Kushwaha B, Srivastava SK, Kumar R, Gopalakrishnan A, Baseer VS, Verma MS.

Characterization of three endemic fish species from Western Ghats using cytogenetic markers. Nucleus 2003;46:110-114.

70. Rishi KK, Mandhan RP. Analysis of C-banded heterochromatin in the chromosomes of *Labeo rohita* (Ham.) (Cyprinidae). CIS 1990;48:3-4.
71. Magtoon W, Arai R. Karyotypes of three cyprinid fishes, *Osteochilus hasselti*, *O. vittatus*, and *Labiobarbus lineatus*, from Thailand. Japan. J. of Ichthyol. 1990;36:483-487. doi: 10.11369/jji1950.36.483
72. Wang RF, He WS, Wu SF. Study on the karyotype and banding of *Semilabeo prochilus* (Pisces). Zool. Res. 1997;18:411-414.
73. Kulabtong S, Suksri S, Nonpayom C, Soonthornkit Y. Rediscovery of the critically endangered cyprinid fish Epalzeorhynchos bicolor (Smith, 1931) from West Thailand (Cypriniformes, Cyprinidae). Biodivers. J. 2014;*5*(2):371-373.
74. Phomikong P, Udduang S, Fukushima M, Srichareondham B, Rattanachamnong D, Jutagate T. Larval fish assemblage patterns in three tributaries of Mekong River in Thailand. Indian J. Fish. 2018;65(2):1-15. doi: 10.21077/ijf.2018.65.2.75121-01
75. Yang JX, Winterbottom R. Phylogeny and zoogeography of the cyprinid Genus *Epalzeorhynchos* Bleeker (Cyprinidae: Ostariophysi). Copeia 1998;1:48-63. doi: 10.2307/1447701
76. Kottelat M. The fishes of the inland waters of Southeast Asia: a catalogue and core bibliography of the fishes known to occur in freshwaters, mangroves and estuaries. Raffles Bull. Zool. 2013;27:1-663.
77. Bertollo LAC, Cioffi MB, Moreira-Filho O. Direct chromosome preparation from freshwater teleost fishes. In: Ozouf-Costaz C, Pisano E, Foresti F, Toledo LFA, editors. Fish cytogenetic techniques: Ray-fin fishes and chondrichthyans. CRC Press; 2015. pp. 21-26.
78. Howell WM, Black DA. 1980. Controlled silver-staining of nucleolus organizer regions with a protective colloidal developer: a 1-step method. Experientia 1980;36:1014-1015.
79. Yano CF, Bertollo LAC, Cioffi MB. Fish-FISH: Molecular cytogenetics in fish species. In: Liehr T, editor. Fluorescence In Situ Hybridization (FISH). Springer; 2017. pp. 429-443.
80. Martins C, Ferreira IA, Oliveira C, Foresti F, Galetti PM. A tandemly repetitive centromeric DNA sequence of the fish *Hoplias malabaricus* (Characiformes: Erythrinidae) is derived from 5S rDNA. Genetica 2006;127:133-141. doi: 10.1007/s10709-005-2674-y.
81. Cioffi MB, Martins C, Centofante L, Jacobina U, Bertollo LAC. Chromosomal variability among allopatric populations of Erythrinidae fish *Hoplias malabaricus*: Mapping of three classes of repetitive DNAs. Cytogene. Genome Res. 2009;125:132-141. doi: 10.1159/000227838.
82. Kubat Z, Hobza R, Vyskot B, Kejnovsky E. Microsatellite accumulation on the Y chromosome in *Silene latifolia*. Genome 2008;51:350-356. doi:10.1139/G08-024.
83. Levan A, Fredga K, Sandberg AA. Nomenclature for centromeric position on chromosomes. Hereditas 1964;52,:201-220. doi: 10.1111/j.1601-5223.1964.tb01953.x
84. Wolf U, Ritter H, Atkin NB, Ohno S. Polyploidization in the fish family Cyprinidae, order Cypriniformes. Humangenetik 1969;7:240-244.
85. Sola L, Gornung E. Classical and molecular cytogenetics of the zebrafish, Danio rerio (Cyprinidae, Cypriniformes): an overview. Genetica 2001;111: 397-412. doi: 10.1023/A:1013776323077
86. Gerbi SA. The evolution of eukaryotic ribosomal DNA. Biosyst. 1986;19(4):247-258. doi: 10.1016/0303-2647(86)90001-8

87. Sochorová J, Garcia S, Gálvez F, Symonová R, Kovařík A. Evolutionary trends in animal ribosomal DNA loci: Introduction to a new online database. Chromosoma 2018;127:141-150. doi: 10.1007/s00412-017-0651-8
88. Khensuwan S, Sassi FMC, Moraes RLR, Jantarat S, Seetapan K, Phintong K, Thongnetr W, Kaewsri S, Jumrusthanasan S, Supiwong W, Rab P, Tanomtong A, Liehr T, Cioffi MB. Chromosomes of Asian Cyprinid Fishes: Genomic Differences in Conserved Karyotypes of 'Poropuntiinae' (Teleostei, Cyprinidae). Animals 2023;13(8):1415. doi: 10.3390/ani13081415
89. Donsakul T, Magtoon W. A chromosome study of three Cyprinid fishes, *Labeo erythrurus*, *L. bicolor* and *L. rohita*. In: Proceedings of the 31st Kasetsart University Annual Conference. Thailand: Kasetsart University; 1993.
90. White MJD. Animal cytology and evolution. 3rd ed. England: Cambridge University Press; 1973.
91. Hoffmann AA, Rieseberg LH. Revisiting the impact of inversions in evolution: from population genetic markers to drivers of adaptive shifts and speciation?. Annu. Rev. Ecol. Evol. Syst. 2008;39:21-42. doi: 10.1146/annurev.ecolsys.39.110707.173532
92. Kirkpatrick M. How and why chromosome inversions evolve. PLoS Biol. 2010;8(9):e1000501. doi: 10.1371/journal.pbio.1000501
93. Charlesworth B, Barton NH. The spread of an inversion with migration and selection. Genetics 2018; 208(1):377-382. doi: 10.1534/genetics.117.300426
94. Wellenreuther M, Bernatchez L. Eco-evolutionary genomics of chromosomal inversions. Trends Ecol. Evol. 2018;33(6):427-440. doi: 10.1016/j.tree.2018.04.002
95. Terêncio ML, Schneider CH, Gross MC, Vicari MR, Farias IP, Passos KB, Feldberg E. Evolutionary dynamics of repetitive DNA in *Semaprochilodus* (Characiformes, Prochilodontidae): a fish model for sex chromosome differentiation. Sex. Dev. 2013;7(6):325-333. doi: 10.1159/000356691
96. Yano CF, Poltronieri J, Bertollo LAC, Artoni RF, Liehr T, Cioffi MB. Chromosomal mapping of repetitive DNAs in *Triportheus trifurcatus* (Characidae, Characiformes): insights into the differentiation of the Z and W chromosomes. PLoS One 2014;9(3):e90946. doi: 10.1371/journal.pone.0090946
97. Cioffi MB, Bertollo LAC, Villa MA, de Oliveira EA, Tanomtong A, Yano CF, Supiwong W, Chaveerach A. Genomic organization of repetitive DNA elements and its implications for the chromosomal evolution of channid fishes (Actinopterygii, Perciformes). PLoS One 2015;10(6):e0130199. doi: 10.1371/journal.pone.0130199
98. Moraes RLR, Bertollo LAC, Marinho MMF, Yano CF, Hatanaka T, Barby FF, Troy WP, Cioffi MB. Evolutionary relationships and cytotaxonomy considerations in the genus *Pyrrhulina* (Characiformes, Lebiasinidae). Zebrafish 2017;14(6):536-546. doi: 10.1089/zeb.2017.1465
99. Moraes RLR, Sember A, Bertollo LAC, De Oliveira EA, Ráb P, Hatanaka T, Marinho MMF, Liehr T, Al-Rikabi ABH, Feldberg E, Viana, PF, Cioffi MB. Comparative cytogenetics and neo-Y formation in small-sized fish species of the genus *Pyrrhulina* (Characiformes, Lebiasinidae). Front. Genet. 2019;10: 678. doi: 10.3389/fgene.2019.00678
100. Sassi FMC, de Oliveira EA, Bertollo LAC, Nirchio M, Hatanaka T, Marinho MMF, Moreira-Filho O, Aroutiounian R, Liehr T. Al-Rikabi ABH, Cioffi, MB. Chromosomal evolution and evolutionary relationships of *Lebiasina* species (Characiformes, Lebiasinidae). Int. J. Mol. Sci. 2019;20(12):2944. doi: 10.3390/ijms20122944
101. Ellegren H. Evolutionary stasis: the stable chromosomes of birds. Trends Ecol. Evol. 2010;25(5):283-

291. doi: 10.1016/j.tree.2009.12.004

102. Zhang X, Zhang S, Zhao Q, Ming R, Tang H. Assembly of allele-aware, chromosomal-scale autopolyploid genomes based on Hi-C data. Nat. plants 2019;5(8):833-845. doi: 10.1038/s41477-019-0487-8

103. Hong Y, Zhou T. Studies on the karyotype and C-banding patterns in *Acheilognathus gracilis* with a discussion on the evolution of acheilognathid fishes. Act. Gen. Sin. 1985;12:143-148.

104. Yu XY, Yu XJ. A schizothoracine fish species, *Diptychus dipogon*, with a very high number of chromosomes. CIS 1990;48:17-18.

Supporting Information: All procedures followed the authorization of the Animal Ethics Committee of KhonKaen University based on the Ethics of Animal Experimentation of the National Research Council of Thailand (Record No. IACUC-KKU-105/63), the animal care and use license for scientific research was U1-07864-2561 (COA.No.MU-IACUC 2019/007) for the fishes of the Mae Klong Basin in Thailand approved from Mahidol University and fish collection authorized by Department of fisheries in Thailand, license number was 49/2564, respectively.

Funding Information: This research project is supported by the National Research Council of Thailand (NCRT): NRCT-RGJ63003-068. The Thailand science research and innovation fund and the University of Phayao (Grant No. FF66-UoE013). PR was funded by Project EXCELLENCE CZ.02.1.01/0.0/0.0/15_003/0000460 OP RDE (PR) and with the institutional support RVO: 67985904 (PR) by the Czech Academy of Sciences. M.d.B.C. was supported by the Conselho Nacional de Desenvolvimento Científico e Tecnológico (CNPq) (302928/2021-9). MBC and TL were supported by Alexander von Humboldt Foundation (Research Group Linkage Programme). We acknowledge support by the German Research Foundation Projekt-Nr. 512648189 and the Open Access Publication Fund of the Thueringer Universitaets- und Landesbibliothek Jena.

Figure 1

Click here to access/download;Figure;Figure 1.tif

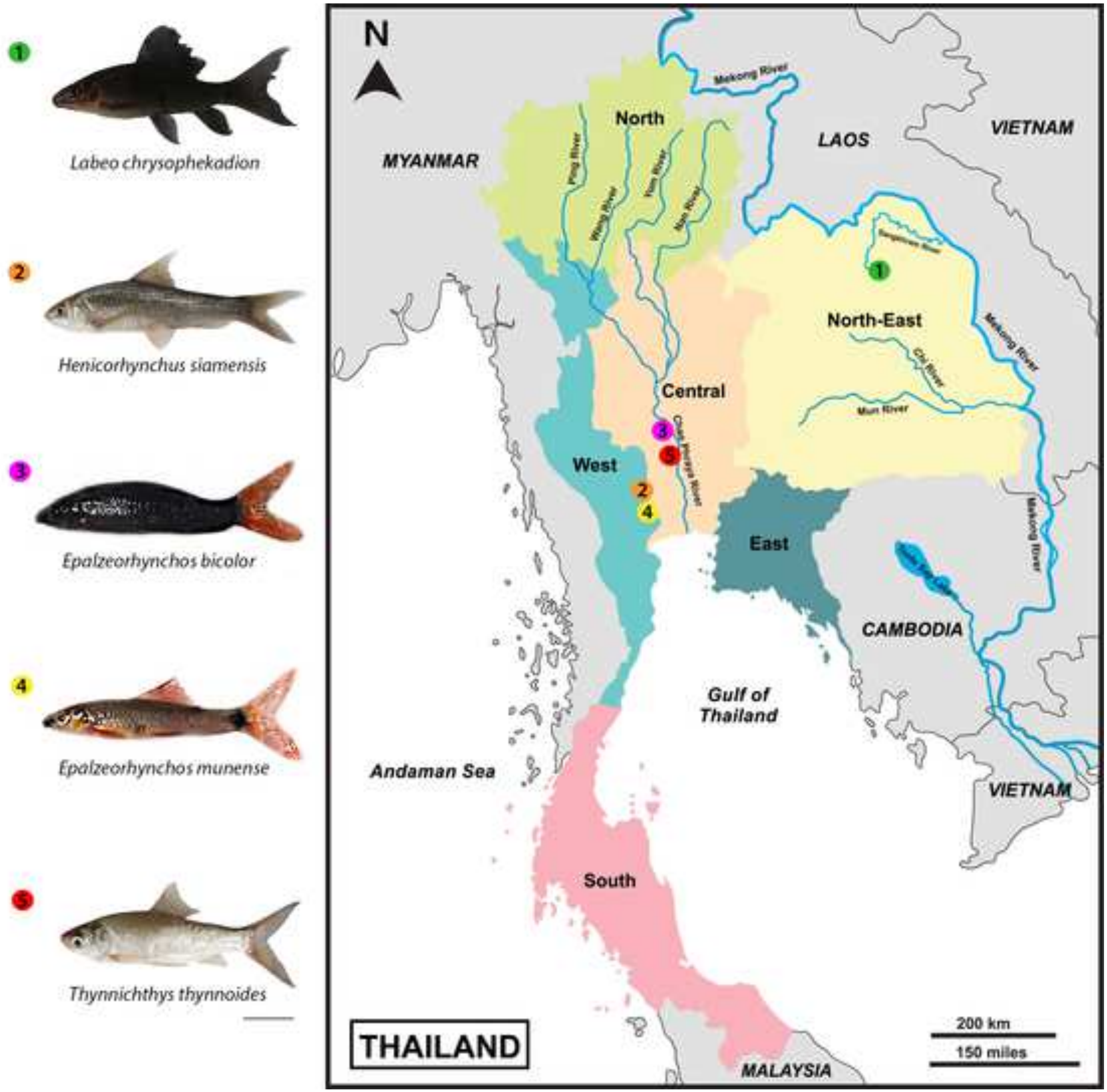

Figure 2

Click here to access/download;Figure;Figure 2.tif

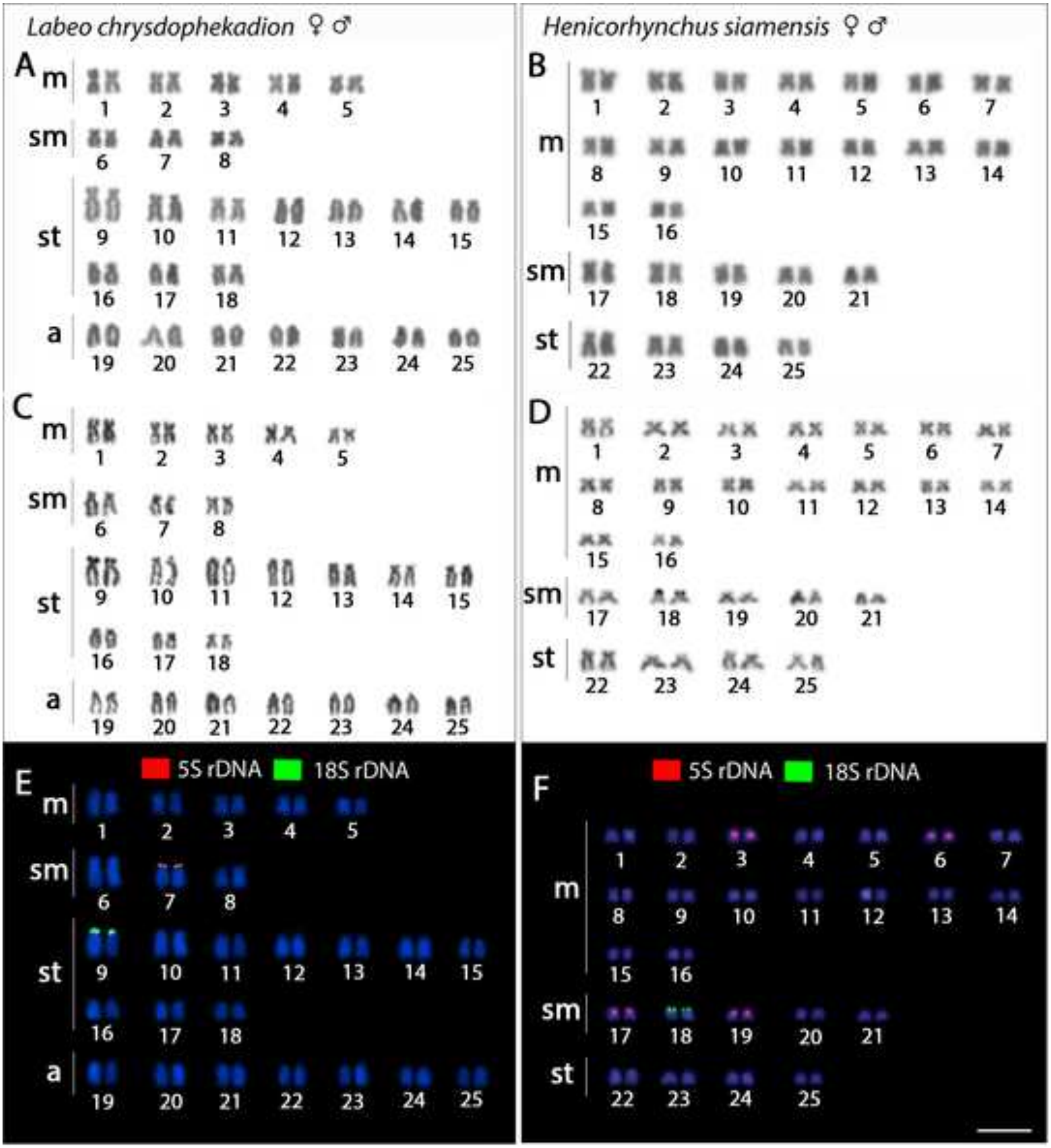

Figure 3

Click here to access/download;Figure;Figure 3.tif

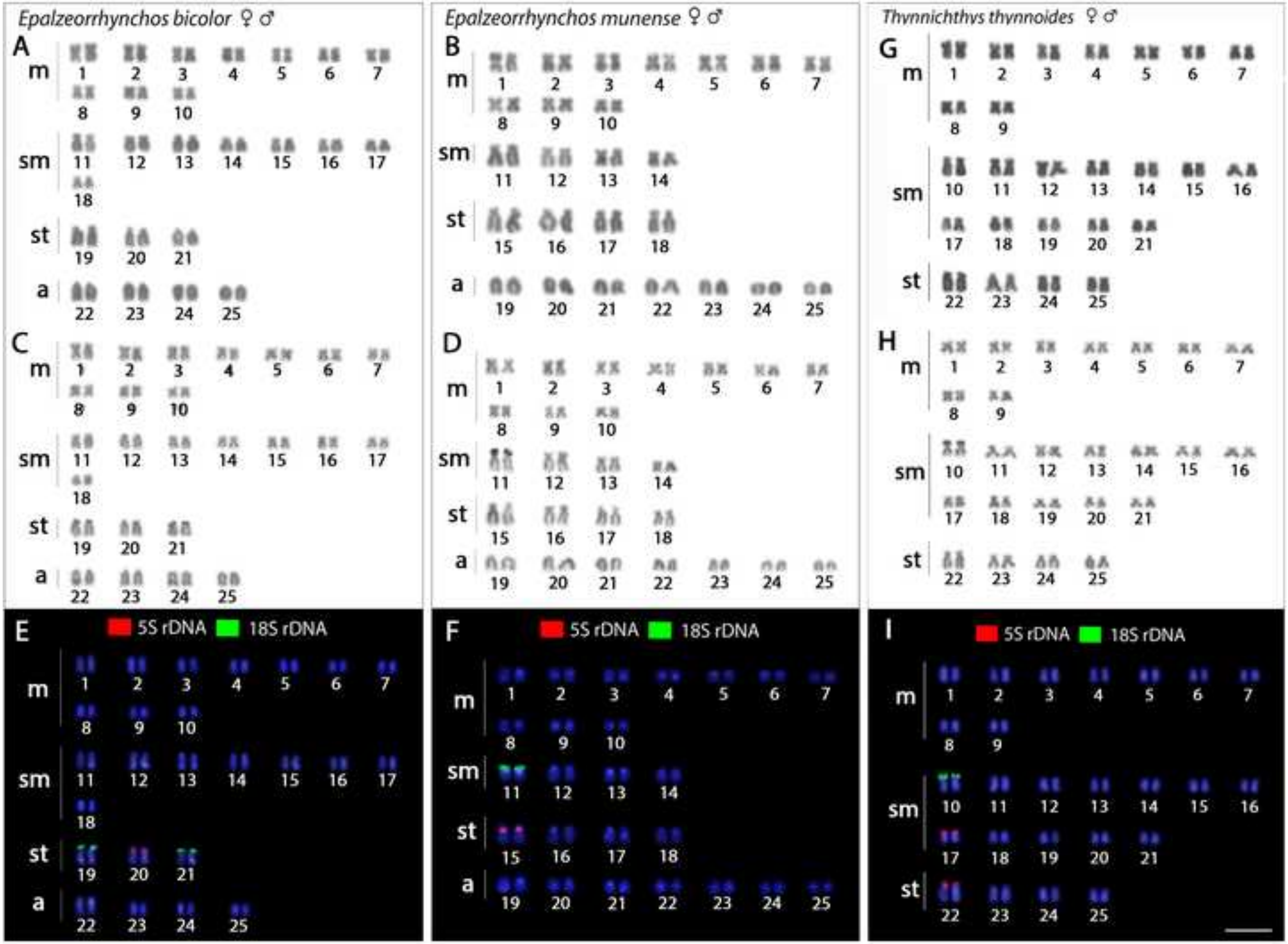

Figure 4

Click here to access/download;Figure;Figure 4.tif

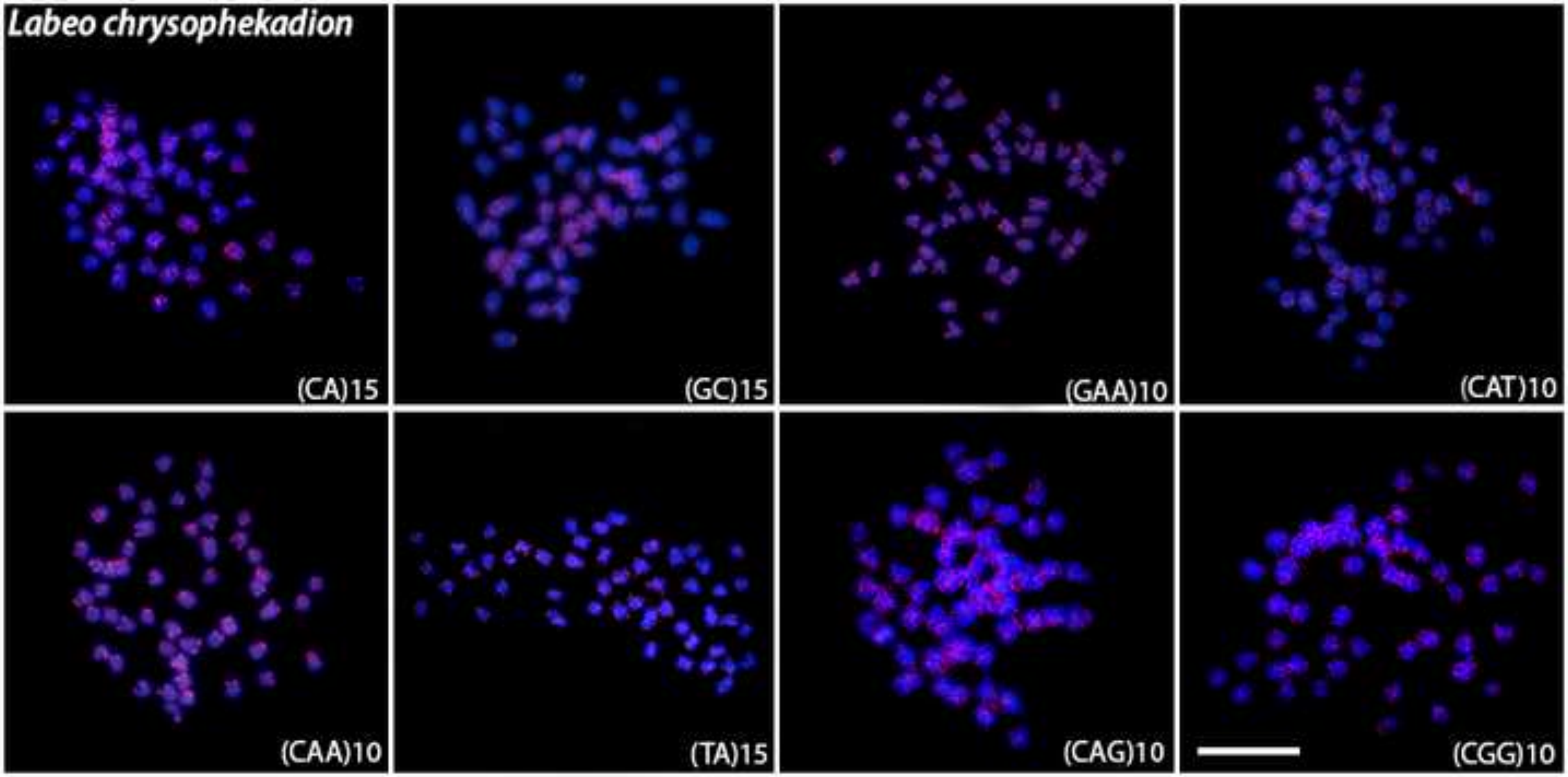

Figure 5

Click here to access/download;Figure;Figure 5.tif

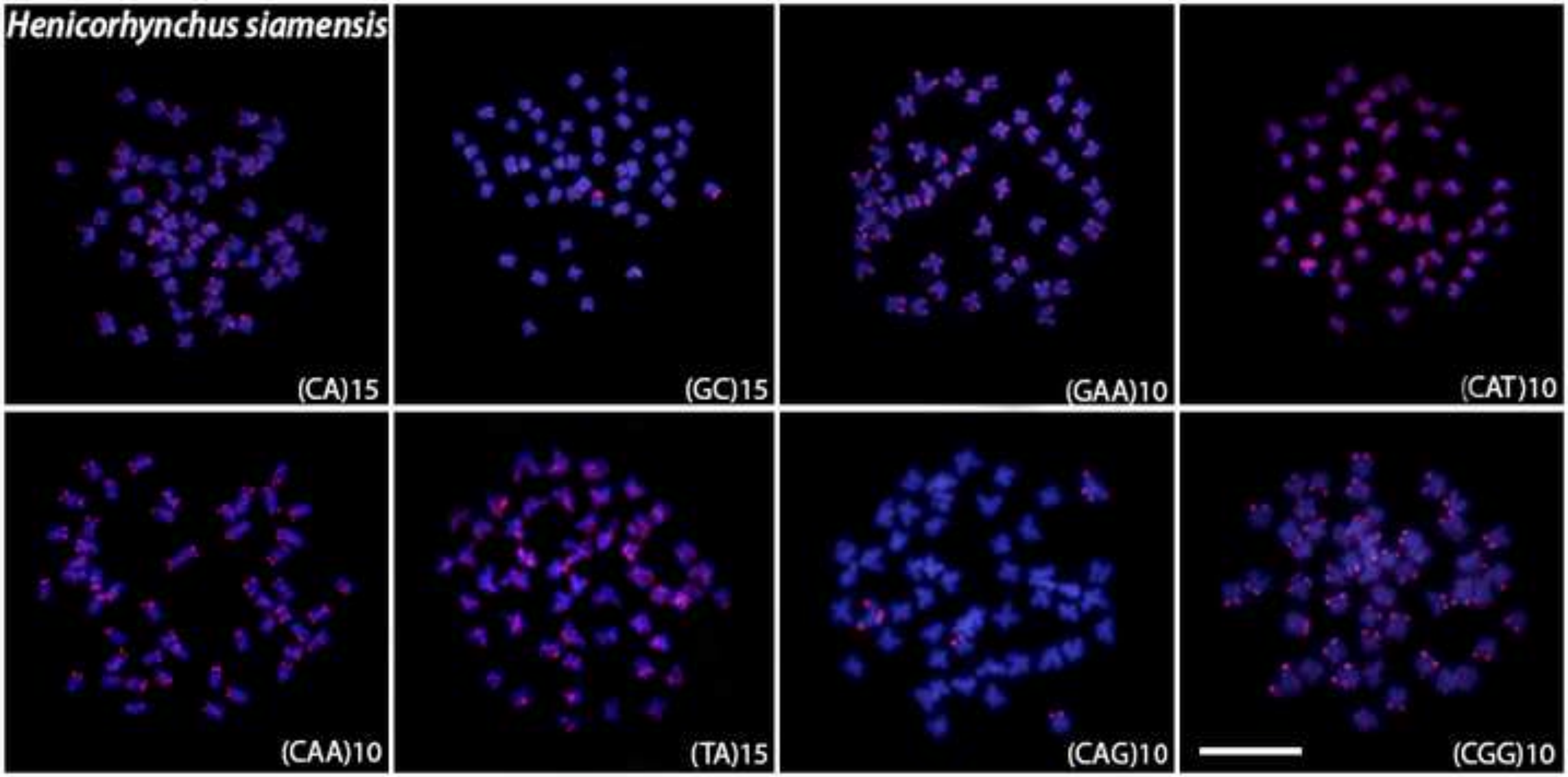

Figure 6

Click here to access/download;Figure;Figure 6.tif

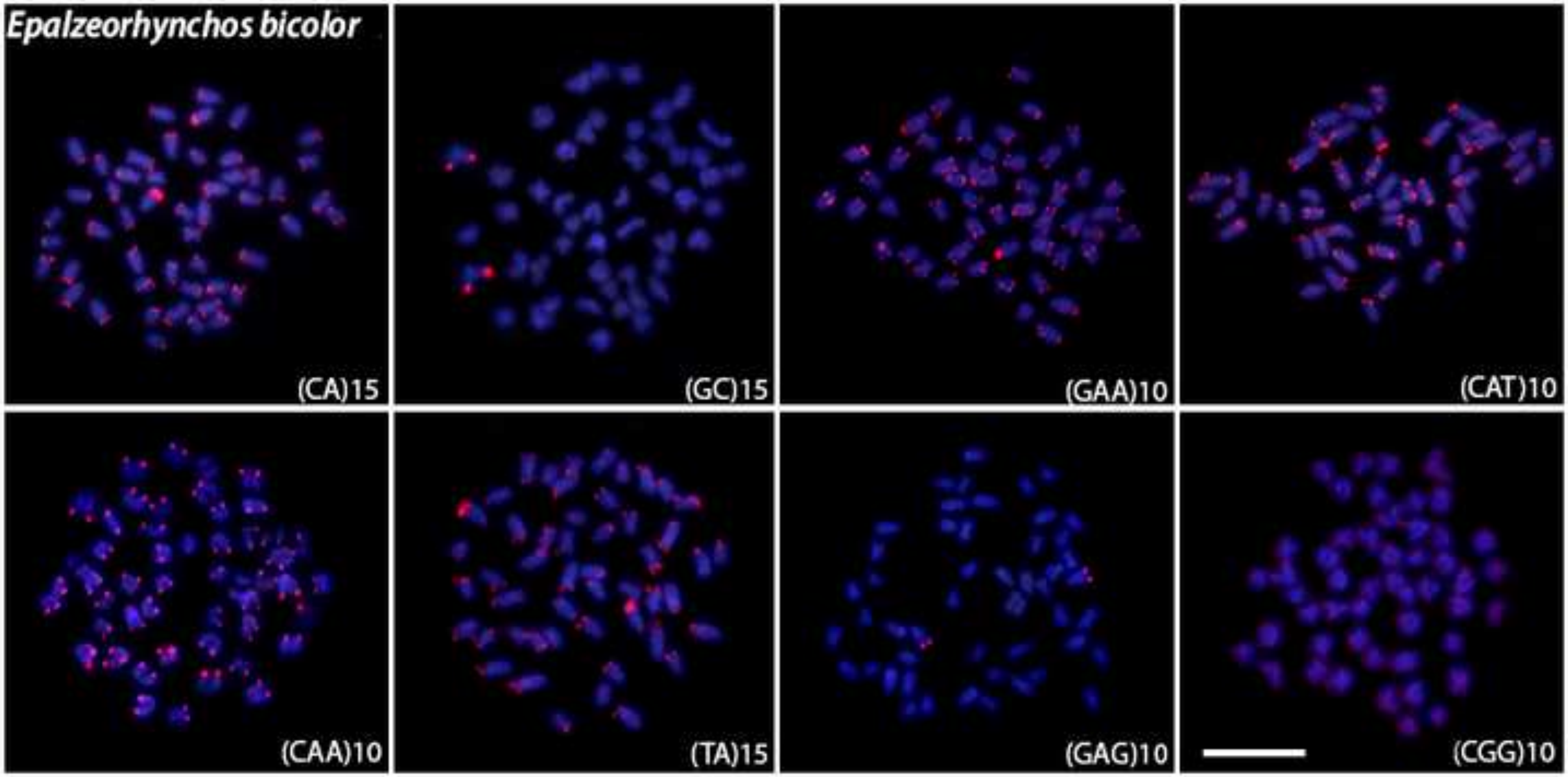

Figure 7

Click here to access/download;Figure;Figure 7.tif

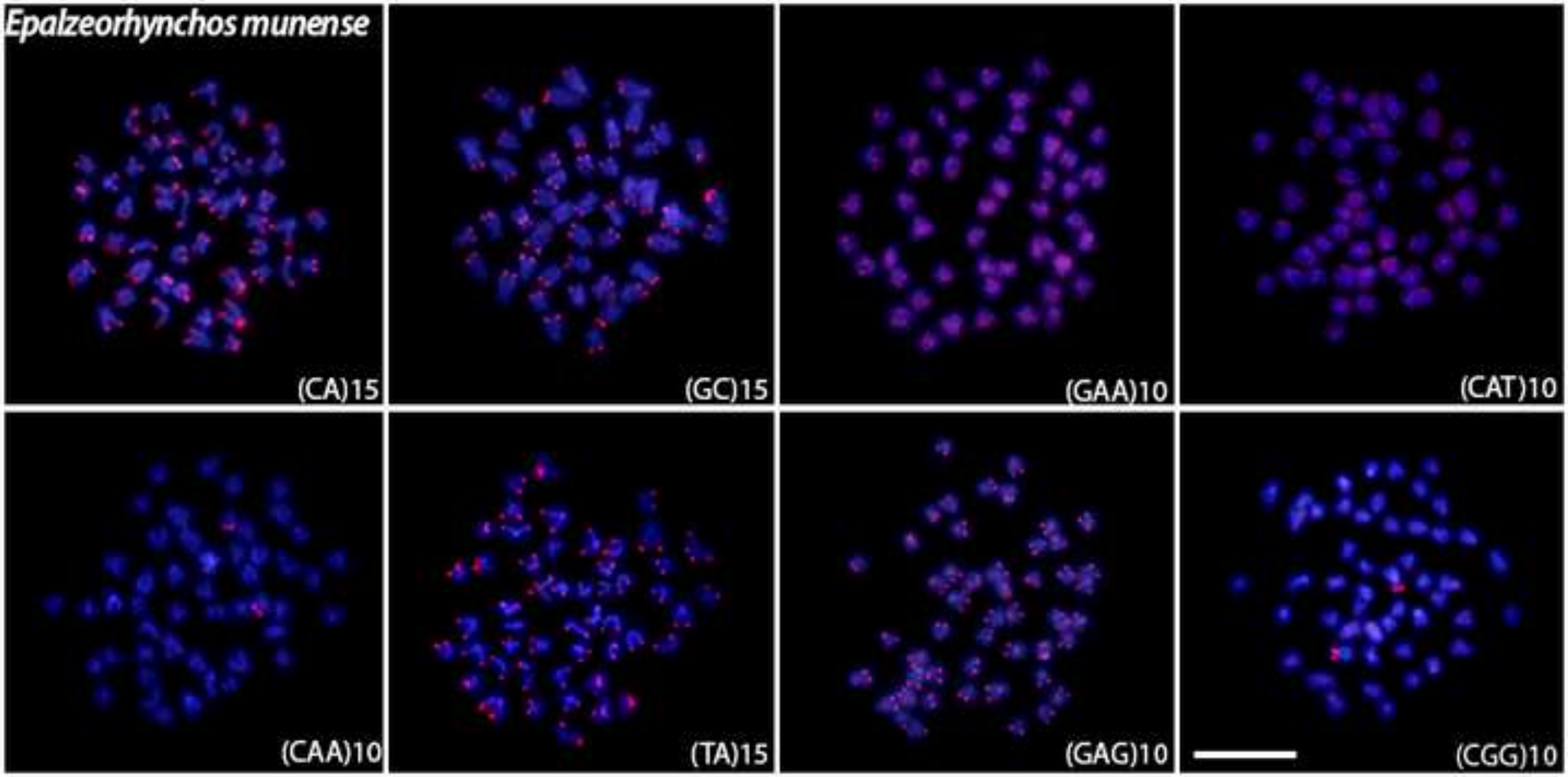

 Click here to access/download;Figure;Figure 8.tif

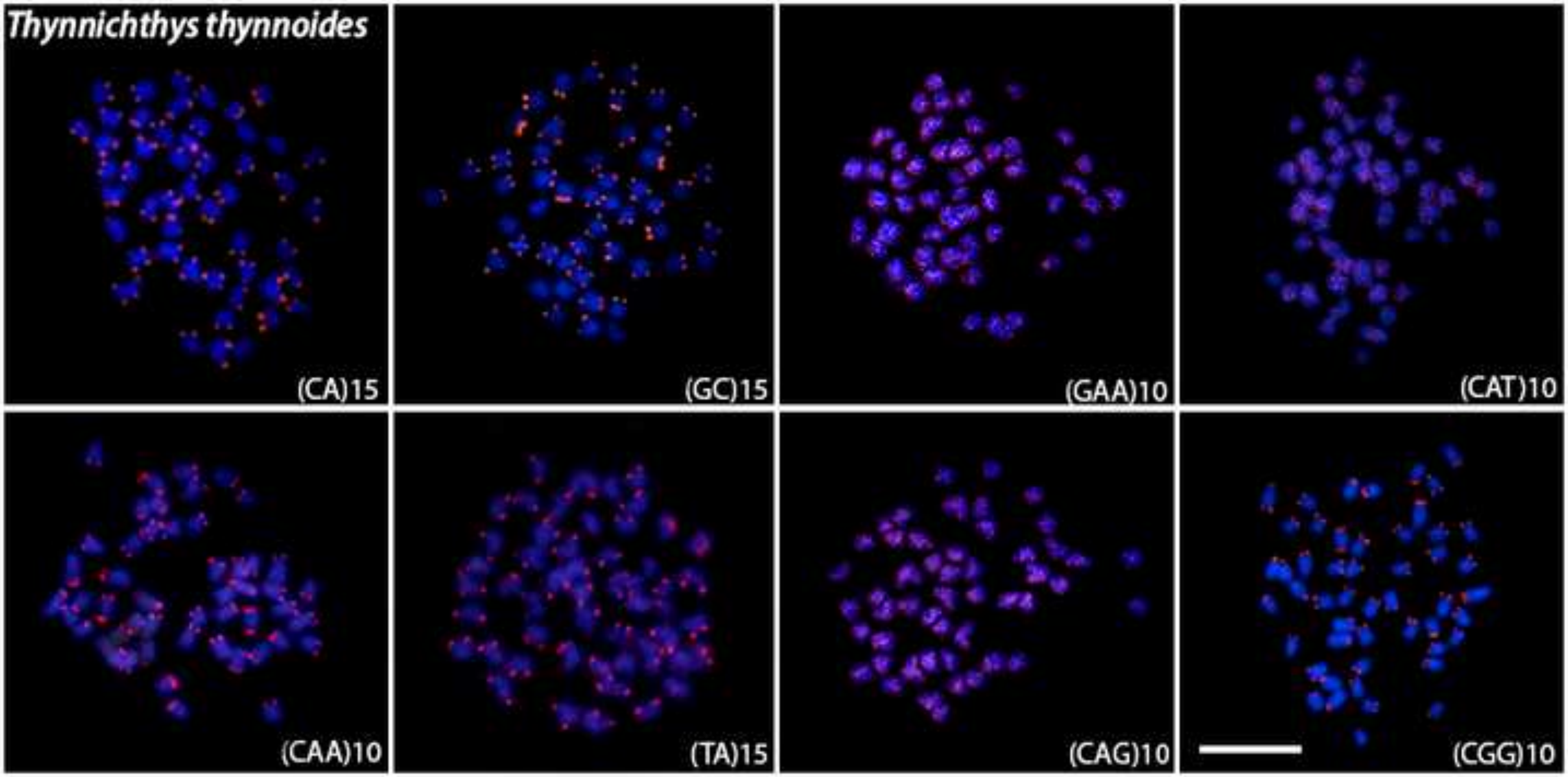

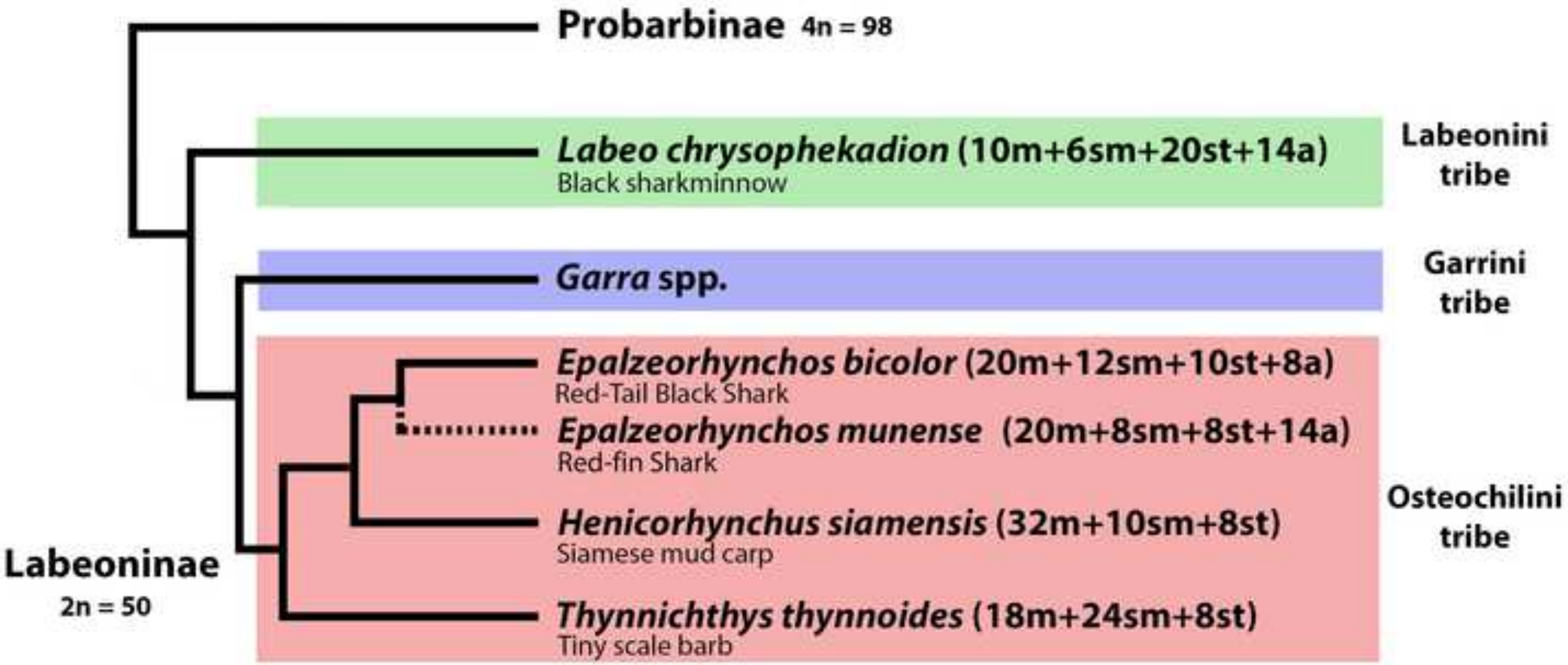
Probarbinae 4n = 98
Labeo chrysophekadion (10m+6sm+20st+14a)
Black sharkminnow
Labeonini tribe
Garra spp.
Garrini tribe
Epalzeorhynchos bicolor (20m+12sm+10st+8a)
Red-Tail Black Shark
Epalzeorhynchos munense (20m+8sm+8st+14a)
Red-fin Shark
Henicorhynchus siamensis (32m+10sm+8st)
Siamese mud carp
Thynnichthys thynnoides (18m+24sm+8st)
Tiny scale barb
Osteochilini tribe
Labeoninae
2n = 50
