# Supplementary material for: Chromosomes of Asian cyprinid fishes: Novel insight into the chromosomal evolution of Labeoninae (Teleostei, Cyprinidae)
Source: PLoS One. 2024 Feb 7;19(2):e0292689. doi: 10.1371/journal.pone.0292689 (PMC10849230; doi:10.1371/journal.pone.0292689)
Supplement: Attachment — Submitted filename: PLOS One comments.doc [file pone.0292689.s001.doc]

**Comments to the authors**

The manuscript (PONE-D-23-14589) entitled ‘**Chromosomes of Asian cyprinid fishes: Novel insight into the chromosomal evolution of Labeoninae (Teleostei, Cyprinidae)’** has been carefully reviewed as per the guidelines of the journal. In my point of view, the manuscript is judged to be of potential interest to the readers and the scientific community as well. While the overall concept of the paper is reasonably satisfactory, it falls within the scope of the journal. However, it has a number of flaws, which need to be addressed before it is formally accepted for publication. For instance, there are some errors in the manuscript's scientific, typographical, and grammatical content. Overall, this manuscript may offer important contributions to the literature, but needs some **major corrections** for possible publication in this journal.

**Title**

Title is long but informative.

**Abstract**

The abstract is concise, factual, and states briefly the purpose of the research. I would recommend suggesting some implications from the findings to represent future prospects of the subject matter. Besides, write full scientific name of *E. munense* as it is in first use.

**Introduction**

The introduction part is poorly written. Rewrite the whole introduction part in order to meet its scientific clarity.

**Materials and Methods**

This section is also written very weak. I suggest consulting any English expert to improve its language quality.

**Results**

All results are ok.

**Discussion**

Although discussion part is somehow linked to the results, but authors need to compare the present results with already published work.

**Figures**

Quality of figure 1, 2 and 3 is not acceptable as the quality of these figures is very poor. So, please revise these figures and change its dpi to 300.

**References**

Crosscheck all references and arrange them in accordance with the guidelines of the journal.

Moreover, the English language of the present version is not up-to-the-mark, and a lot of mistakes and syntaxes have been seen in the manuscript. It is firmly recommended to reconsider the English and get the manuscript to be checked by a professional editor
