# Supplementary material for: Chromosomes of Asian cyprinid fishes: Novel insight into the chromosomal evolution of Labeoninae (Teleostei, Cyprinidae)
Source: PLoS One. 2024 Feb 7;19(2):e0292689. doi: 10.1371/journal.pone.0292689 (PMC10849230; doi:10.1371/journal.pone.0292689)
Supplement: Attachment — Submitted filename: Response to Reviewers.docx [file pone.0292689.s003.docx]

**June 22^nd^, 2023**

## Dear Editor of **PLOS One,**

We would like to submit the revised version of the manuscript entitled “**Chromosomes of Asian cyprinid fishes: Novel insight into the chromosomal evolution of Labeoninae (Teleostei, Cyprinidae)** to be considered for publication as an original article in **PLOS One.**

An itemized list of all changes made, in response to comments from the Editor and reviewers, is given below and all the changes performed are highlighted as track changes in the revised manuscript (attached). We appreciate the contributions pointed out by reviewers and the editor.

**Editorial Comments**

When submitting your revision, we need you to address these additional requirements.

1. Please ensure that your manuscript meets PLOS ONE's style requirements, including those for file naming. The PLOS ONE style templates can be found at 
<https://journals.plos.org/plosone/s/file?id=wjVg/PLOSOne_formatting_sample_main_body.pdf> and 
<https://journals.plos.org/plosone/s/file?id=ba62/PLOSOne_formatting_sample_title_authors_affiliations.pdf>

**We modified the files according to the templates.**

2. To comply with PLOS ONE submissions requirements, in your Methods section, please provide additional information regarding the experiments involving animals and ensure you have included details on (1) methods of sacrifice, (2) methods of anesthesia and/or analgesia, and (3) efforts to alleviate suffering.

**This information was added in the sampling subsection.**

3. We suggest you thoroughly copyedit your manuscript for language usage, spelling, and grammar. If you do not know anyone who can help you do this, you may wish to consider employing a professional scientific editing service.

**We copyedit the entire manuscript and changes are indicated in the revised manuscript file.**

Whilst you may use any professional scientific editing service of your choice, PLOS has partnered with both American Journal Experts (AJE) and Editage to provide discounted services to PLOS authors. Both organizations have experience helping authors meet PLOS guidelines and can provide language editing, translation, manuscript formatting, and figure formatting to ensure your manuscript meets our submission guidelines. To take advantage of our partnership with AJE, visit the AJE website (<http://learn.aje.com/plos/>) for a 15% discount off AJE services. To take advantage of our partnership with Editage, visit the Editage website ([www.editage.com](http://www.editage.com)) and enter referral code PLOSEDIT for a 15% discount off Editage services.  If the PLOS editorial team finds any language issues in text that either AJE or Editage has edited, the service provider will re-edit the text for free.

Upon resubmission, please provide the following: 
● The name of the colleague or the details of the professional service that edited your manuscript
● A copy of your manuscript showing your changes by either highlighting them or using track changes (uploaded as a *supporting information* file)
● A clean copy of the edited manuscript (uploaded as the new *manuscript* file)

4. We noticed you have some minor occurrences of overlapping text with the following previous publication(s), which needs to be addressed:

<https://www.scielo.br/j/gmb/a/FYWGsxSngHdjW9B7HWrBZfw/?lang=en>
<https://www.mdpi.com/2076-2615/13/8/1415>

In your revision, cite all your sources (including your own works), and quote or rephrase any duplicated text outside the methods section. A further consideration is dependent on these concerns being addressed.

**We modified the manuscript to avoid overlapping with the abovementioned sections.**

5.In your Methods section, please provide additional information regarding the permits you obtained for the work. Please ensure you have included the full name of the authority that approved the field site access and, if no permits were required, a brief statement explaining why.

**Such information is described in the sampling subsection. “All procedures followed the authorization of the Animal Ethics Committee of KhonKaen University based on the Ethics of Animal Experimentation of the National Research Council of Thailand (Record No. IACUC-KKU-105/63), the animal care and use license for scientific research was U1-07864-2561 (COA.No.MU-IACUC 2019/007) for the fishes of the Mae Klong Basin in Thailand approved from Mahidol University and fish collection authorized by Department of fisheries in Thailand, license number was 49/2564, respectively.”**

6. Thank you for stating the following in the Acknowledgments Section of your manuscript: 
   "This research project is supported by the National Research Council of Thailand (NCRT):NRCT-RGJ63003-068. The Thailand science research and innovation fund and the University of Phayao (Grant No. FF66-UoE013)"

We note that you have provided funding information that is not currently declared in your Funding Statement. However, funding information should not appear in the Acknowledgments section or other areas of your manuscript. We will only publish funding information present in the Funding Statement section of the online submission form. 
Please remove any funding-related text from the manuscript and let us know how you would like to update your Funding Statement. Currently, your Funding Statement reads as follows: 
  "This research project is supported by the National Research Council of Thailand (NCRT): NRCT-RGJ63003-068. The Thailand science research and innovation fund and the University of Phayao (Grant No. FF66-UoE013)."

Please include your amended statements within your cover letter; we will change the online submission form on your behalf.

**We removed the Acknowledgements section in the revised manuscript. The correct funding information is the one indicated in the next question.**

7. Thank you for stating the following financial disclosure: 
   "This research project is supported by the National Research Council of Thailand (NCRT): NRCT-RGJ63003-068. The Thailand science research and innovation fund and the University of Phayao (Grant No. FF66-UoE013)."

Please state what role the funders took in the study.  If the funders had no role, please state: "The funders had no role in study design, data collection and analysis, decision to publish, or preparation of the manuscript." 
If this statement is not correct you must amend it as needed. 
Please include this amended Role of Funder statement in your cover letter; we will change the online submission form on your behalf.

**Role of Funder:**

**"The funders had no role in study design, data collection and analysis, decision to publish, or preparation of the manuscript."**

8. Your ethics statement should only appear in the Methods section of your manuscript. If your ethics statement is written in any section besides the Methods, please delete it from any other section.

**We removed this information and the whole Supporting Information section.**

9. We note that Figure 1 in your submission contain [map/satellite] images which may be copyrighted. All PLOS content is published under the Creative Commons Attribution License (CC BY 4.0), which means that the manuscript, images, and Supporting Information files will be freely available online, and any third party is permitted to access, download, copy, distribute, and use these materials in any way, even commercially, with proper attribution. For these reasons, we cannot publish previously copyrighted maps or satellite images created using proprietary data, such as Google software (Google Maps, Street View, and Earth). For more information, see our copyright guidelines: <http://journals.plos.org/plosone/s/licenses-and-copyright>.
 
We require you to either (a) present written permission from the copyright holder to publish these figures specifically under the CC BY 4.0 license, or (b) remove the figures from your submission:

**This map was created by our research group using only copyright-free data available on QGIS.**  
a. You may seek permission from the original copyright holder of Figure 1 to publish the content specifically under the CC BY 4.0 license.  
 
We recommend that you contact the original copyright holder with the Content Permission Form (<http://journals.plos.org/plosone/s/file?id=7c09/content-permission-form.pdf>) and the following text:
“I request permission for the open-access journal PLOS ONE to publish XXX under the Creative Commons Attribution License (CCAL) CC BY 4.0 (<http://creativecommons.org/licenses/by/4.0/>). Please be aware that this license allows unrestricted use and distribution, even commercially, by third parties. Please reply and provide explicit written permission to publish XXX under a CC BY license and complete the attached form.”
 
Please upload the completed Content Permission Form or other proof of granted permissions as an "Other" file with your submission.
 
In the figure caption of the copyrighted figure, please include the following text: “Reprinted from [ref] under a CC BY license, with permission from [name of publisher], original copyright [original copyright year].”
 
b. If you are unable to obtain permission from the original copyright holder to publish these figures under the CC BY 4.0 license or if the copyright holder’s requirements are incompatible with the CC BY 4.0 license, please either i) remove the figure or ii) supply a replacement figure that complies with the CC BY 4.0 license. Please check copyright information on all replacement figures and update the figure caption with source information. If applicable, please specify in the figure caption text when a figure is similar but not identical to the original image and is therefore for illustrative purposes only.
The following resources for replacing copyrighted map figures may be helpful:
 
USGS National Map Viewer (public domain): <http://viewer.nationalmap.gov/viewer/>
The Gateway to Astronaut Photography of Earth (public domain): <http://eol.jsc.nasa.gov/sseop/clickmap/>
Maps at the CIA (public domain): <https://www.cia.gov/library/publications/the-world-factbook/index.html> and <https://www.cia.gov/library/publications/cia-maps-publications/index.html>
NASA Earth Observatory (public domain): <http://earthobservatory.nasa.gov/>
Landsat: <http://landsat.visibleearth.nasa.gov/>
USGS EROS (Earth Resources Observatory and Science (EROS) Center) (public domain): [http://eros.usgs.gov/#](http://eros.usgs.gov/)
Natural Earth (public domain): <http://www.naturalearthdata.com/>

10. Please include a copy of Table 2 which you refer to in your text on page 4.

**Table included in the revised manuscript files.**

**Reviewer 1**

The manuscript (PONE-D-23-14589) entitled ‘**Chromosomes of Asian cyprinid fishes: Novel insight into the chromosomal evolution of Labeoninae (Teleostei, Cyprinidae)’** has been carefully reviewed as per the guidelines of the journal. In my point of view, the manuscript is judged to be of potential interest to the readers and the scientific community as well. While the overall concept of the paper is reasonably satisfactory, it falls within the scope of the journal. However, it has a number of flaws, which need to be addressed before it is formally accepted for publication. For instance, there are some errors in the manuscript's scientific, typographical, and grammatical content. Overall, this manuscript may offer important contributions to the literature, but needs some **major corrections** for possible publication in this journal.

**Title**

Title is long but informative.

**Abstract**

The abstract is concise, factual, and states briefly the purpose of the research. I would recommend suggesting some implications from the findings to represent future prospects of the subject matter. Besides, write full scientific name of *E. munense* as it is in first use.

**We performed the modifications as suggested.**

**Introduction**

The introduction part is poorly written. Rewrite the whole introduction part in order to meet its scientific clarity.

**We have rewritten great part of the Introduction to make it more clear, following the reviewer request.**

**Materials and Methods**

This section is also written very weak. I suggest consulting any English expert to improve its language quality.

**We performed a grammar checking in the whole manuscript.**

**Results**

All results are ok.

**Discussion**

Although discussion part is somehow linked to the results, but authors need to compare the present results with already published work.

**Our discussion section compared the herein obtained results with previous published works**

**Figures**

Quality of figure 1, 2 and 3 is not acceptable as the quality of these figures is very poor. So, please revise these figures and change its dpi to 300.

**Modifications performed as suggested.**

**References**

Crosscheck all references and arrange them in accordance with the guidelines of the journal.

Moreover, the English language of the present version is not up-to-the-mark, and a lot of mistakes and syntaxes have been seen in the manuscript. It is firmly recommended to reconsider the English and get the manuscript to be checked by a professional editor

**We performed a copyedit in the whole text to fit in the suggested criteria.**

**Reviewer 2**

1.There are typographical errors. Results should be in past tense and discussion should be in present tense.

**We checked the whole text in the revised version.**

2. More details should be added for molecular results in the discussion and should be compared with earlier work.

**We modified the discussion section to include such information.**

3. Explanation of figures should be added under the figures not in the text.

**We added those informations on the figure legends.**

4. Results of molecular studies with different probes should be compared with respect to each species because all the results compiled in one figure for each species. So, it is difficult to compared the results.

**We understand that a single figure comparing all species can be better to compare the results. However, we are afraid that chromosomes will be very small in the publication and no clear patterns will be observed. To solve this, we described better the general trends and arranged the figures to appear in sequence on the published version.**

5. Figure numbers for chromosome plates whether A/B/C should be added in the text.

**We included this information in the revised version.**

6. There are 104 references but these are not properly discussed in the text.

**We appreciate the comments but in fact we believe that all references are properly addressed in the main text, including the discussion section. Moreover, we re-analyzed this point in the revised version and also checked for possible mistakes in references.**

7. The localization of 5S or 18S RNA should be marked on the plates with arrows to confirm their presence. same with the results of different probes.

**We included arrows to clarify the FISH signals. On scattered patterns we choose to do not include because they can be difficult to properly address the position.**

8. The data is very less for phylogenetic analysis. more data should be added to draw evolutionary relationships based on phylogenetic tree to justify the title of the paper.

**We understand that the title does not infer about phylogenetic tree. We discussed the chromosomal data in an evolutionary context, based on previous phylogenetic trees.**

Thank you for your kind consideration of this paper.

Yours sincerely,

**Weerayuth Supiwong**

Corresponding author
